# The Bohl Spectrum for Linear Nonautonomous Differential Equations

**DOI:** 10.1007/s10884-016-9530-x

**Published:** 2016-04-07

**Authors:** Thai Son Doan, Kenneth J. Palmer, Martin Rasmussen

**Affiliations:** 1 Department of Probability and Statistics, Institute of Mathematics, Vietnam Academy of Science and Technology, Hanoi, Vietnam; 2 Department of Mathematics, National Taiwan University, No. 1, Sec. 4, Roosevelt Road, Taipei, 106 Taiwan; 3 Department of Mathematics, Imperial College London, 180 Queen’s Gate, London, SW7 2AZ UK

**Keywords:** Bohl exponent, Bohl spectrum, Lyapunov exponent, Nonautonomous linear differential equation, Sacker–Sell spectrum, 34A30, 34D05, 37H15

## Abstract

We develop the Bohl spectrum for nonautonomous linear differential equations on a half line, which is a spectral concept that lies between the Lyapunov and the Sacker–Sell spectra. We prove that the Bohl spectrum is given by the union of finitely many intervals, and we show by means of an explicit example that the Bohl spectrum does not coincide with the Sacker–Sell spectrum in general even for bounded systems. We demonstrate for this example that any higher-order nonlinear perturbation is exponentially stable (which is not evident from the Sacker–Sell spectrum), but we show that in general this is not true. We also analyze in detail situations in which the Bohl spectrum is identical to the Sacker–Sell spectrum.

## Introduction

The stability theory for linear nonautonomous differential equations has its origin in A.M. Lyapunov’s celebrated PhD Thesis [20], where he introduces characteristic numbers, so-called *Lyapunov exponents*, which are given by accumulation points of exponential growth rates of individual solutions. It is well-known that in case of negative Lyapunov exponents, the stability of nonlinearly perturbed systems is not guaranteed without an additional regularity condition.

In the 1970s, R.S. Sacker and G.R. Sell developed the Sacker–Sell spectrum theory for nonautonomous differential equations. In contrast to the Lyapunov spectrum, the Sacker–Sell spectrum is not a solution-based spectral theory, but rather is based on the concept of an exponential dichotomy, which concerns uniform growth behavior in subspaces and extends the idea of hyperbolicity to explicitly time-dependent systems. If the Sacker–Sell spectrum lies left of zero, then the uniform exponential stability of nonlinearly perturbed systems is guaranteed.

It was shown in [22] that the regularity condition on Lyapunov exponents can be more robustly replaced by a nonuniform exponential dichotomy. Here the nonuniformity refers to time, and in contrast to that, so-called Bohl exponents, introduced by Bohl [11], measure exponential growth along solutions uniformly in time. Bohl exponents have been studied extensively in the literature [14], and current research focuses on applications to differential-algebraic equations and control theory [2, 10, 17, 19, 30], and parabolic partial differential equations [23]. In this paper, we develop the Bohl spectrum as union of all possible Bohl exponents of a nonautonomous linear differential equation on a half line. We show that the Bohl spectrum lies between the Lyapunov and the Sacker–Sell spectrum and that the Bohl spectrum is given by the union of finitely many (not necessarily closed) intervals. Each Bohl spectral interval is associated with a linear subspace, leading to a filtration of subspaces which is finer than the filtration obtained by the Sacker–Sell spectrum.

We show by means of an explicit example that the Bohl spectrum can be a proper subset of the Sacker–Sell spectrum even if the system is bounded. We analyze in detail situations in which the Bohl spectrum is identical to the Sacker–Sell spectrum, and in particular, we obtain this for bounded diagonalizable systems, integrally separated systems, and systems with Sacker–Sell point spectrum. The fact that the Bohl and Sacker–Sell spectra coincide for diagonalizable systems shows that the Bohl spectrum mainly gives information about the asymptotic behaviour of individual solutions whereas the Sacker–Sell also embodies information about the relation between different solutions, in particular, whether or not the angle between solutions is bounded below by a positive number. An interesting problem in this context is to give necessary and sufficient conditions that the Bohl and Sacker–Sell spectra coincide.

The example referred to above shows that the Sacker–Sell spectrum can extend past zero even when the Bohl spectrum is given by a negative number. We demonstrate for this example that any higher-order nonlinear perturbation is exponentially stable, although this is not evident from the Sacker–Sell spectrum. In the last section of this paper, we discuss an example with negative Bohl spectrum such that for a certain nonlinear perturbation, the perturbed system is unstable. This means that it is not possible to prove in general that if the Bohl spectrum lies to the left of zero, then any higher-order nonlinear perturbation is exponentially stable. In a forthcoming paper, we will provide additional conditions on the nonlinearities which give a positive answer to this question, even in situations where the Sacker–Sell spectrum intersects the positive half axis.

This paper is organized as follows. In Sect. 2, we provide basic material on the Lyapunov and Sacker–Sell spectrum, and in Sect. 3, we introduce the Bohl spectrum. Section 4 is devoted to prove the Spectral Theorem, which says that the Bohl spectrum is given by the union of finitely many intervals. We compare the Bohl spectrum and the Sacker–Sell spectrum in Sect. 5, and we discuss nonlinear perturbations to linear systems with negative Bohl spectrum in Sect. 6.

## Lyapunov and Sacker–Sell Spectrum

In this section, we review the definition and basic properties of the two main spectral concepts for nonautonomous differential equations: the Lyapunov spectrum and the Sacker–Sell spectrum.

We consider a linear nonautonomous differential equation of the form

1 $$\begin{aligned} \dot{x}=A(t)x\,, \end{aligned}$$

where $A:\mathbb {R}_{0}^{+}\rightarrow \mathbb {R}^{d\times d}$ is a locally integrable matrix-valued function, i.e. for any $0\le a < b$, we have $\int _a^b \Vert A(t)\Vert \,\mathrm {d}t<\infty$. Let $X:\mathbb {R}_0^+\rightarrow \mathbb {R}^{d\times d}$ be the *fundamental matrix* of (1), i.e. $X(\cdot )\xi$ solves (1) with the initial value condition $x(0)=\xi$, where $\xi \in \mathbb {R}^d$.

The Lyapunov spectrum describes asymptotic growth of individual solutions of (1).

### **Definition 1**

(*Lyapunov spectrum*) The *lower* and *upper characteristic Lyapunov exponents* of a particular non-zero solution $X(\cdot )\xi$ of (1) are defined by

$$\begin{aligned} \chi _-(\xi ):=\liminf _{t\rightarrow \infty }\frac{1}{t}\ln \Vert X(t)\xi \Vert \end{aligned}$$

and

$$\begin{aligned} \chi _+(\xi ):=\limsup _{t\rightarrow \infty }\frac{1}{t}\ln \Vert X(t)\xi \Vert \,. \end{aligned}$$

The *Lyapunov spectrum of* (1) is then defined as

$$\begin{aligned} \Sigma _\mathrm{Lya}:=\bigcup _{\xi \in \mathbb {R}^d\setminus \{0\}} \{\chi _+(\xi )\}\,. \end{aligned}$$

It is well-known [1, 6] that there exist $n\in \{1,\dots ,d\}$ and $\xi _1,\dots ,\xi _n\in \mathbb {R}^d\setminus \{0\}$ such that

$$\begin{aligned} \Sigma _\mathrm{Lya} = \bigcup _{i=1}^n \,\{\chi _+(\xi _i)\} \,. \end{aligned}$$

In contrast to the Lyapunov spectrum, the Sacker–Sell spectrum is based on a hyperbolicity concept for nonautonomous differential equations, given by an exponential dichotomy.

### **Definition 2**

(*Exponential dichotomy*) The linear differential equation (1) admits an *exponential dichotomy* with growth rate $\gamma \in \mathbb {R}$ if there exist a projector $P\in \mathbb {R}^{d\times d}$, and constants $K\ge 1$ and $\alpha >0$, such that

$$\begin{aligned} \Vert X(t)P X^{-1}(s)\Vert\le & {} K e^{(\gamma -\alpha )(t-s)} \quad \text {for all }\,0\le s \le t\,,\\ \Vert X(t)(\mathbbm {1}-P) X^{-1}(s)\Vert\le & {} K e^{(\gamma +\alpha )(t-s)} \quad \text {for all }\,0\le t \le s\,, \end{aligned}$$

where $\mathbbm {1}$ denotes the unit matrix. In addition, we say that (1) admits an *exponential dichotomy* with growth rate $\infty$ if there exists a $\gamma \in \mathbb {R}$ such that (1) admits an *exponential dichotomy* with growth rate $\gamma$ and projector $P=\mathbbm {1}$, and (1) is said to admit an *exponential dichotomy* with growth rate $-\infty$ if there exists a $\gamma \in \mathbb {R}$ such that (1) admits an *exponential dichotomy* with growth rate $\gamma$ and projector $P=0$, the zero matrix.

The range of the projector *P* of an exponential dichotomy is called the *pseudo-stable space*, and the null space of the projector *P* is called a *pseudo-unstable space*. Note that in contrast to the pseudo-unstable space, the pseudo-stable space is uniquely determined for exponential dichotomies on $\mathbb {R}^+_0$ [27].

The Sacker–Sell spectrum is then given by set of all growth rates $\gamma$ such that the linear system does not admit an exponential dichotomy with growth rate $\gamma$.

### **Definition 3**

(*Sacker–Sell spectrum*) The *Sacker–Sell spectrum* of the linear differential equation (1) is defined by

$$\begin{aligned} \Sigma _\mathrm{SS} := \{\gamma \in \overline{\mathbb {R}}:&(1) \hbox { does not admit an exponential dichotomy}\\&\hbox { with growth rate } \gamma \}\,, \end{aligned}$$

where $$\overline{\mathbb {R}}:= \mathbb {R}\cup \{-\infty ,\infty \}$$.

The Sacker–Sell spectrum was introduced by Sacker and Sell in [28] for skew product flows with compact base. It was generalized to nonautonomous dynamical systems with not necessarily compact base in [3, 29] and for systems defined on a half-line in [27].

The Spectral Theorem (see [18, 27] for the half-line case) describes the structure of the dichotomy spectrum.

### **Theorem 4**

(Sacker–Sell Spectral Theorem) For the linear differential equation (1), there exists a $k\in \{1,\ldots , d\}$ such that

$$\begin{aligned} \Sigma _\mathrm{SS} = [a_1, b_1]\cup \cdots \cup [a_k,b_k] \end{aligned}$$

with $-\infty \le a_1\le b_1<a_2\le b_2< \cdots <a_k\le b_k\le \infty$. In addition, there exists a corresponding filtration

$$\begin{aligned} \{0\}={\mathcal {W}}_0 \subsetneq {\mathcal {W}}_1\subsetneq {\mathcal {W}}_2\subsetneq \dots \subsetneq {\mathcal {W}}_k =\mathbb {R}^d\,, \end{aligned}$$

which satisfies the dynamical characterization

$$\begin{aligned} {\mathcal {W}}_i= \Big \{\textstyle \xi \in \mathbb {R}^d: \sup _{t\in \mathbb {R}^+_0} \Vert X(t)\xi \Vert e^{-\gamma t} < \infty \Big \} \end{aligned}$$

for all $i\in \{1,\dots ,k-1\}$ and $\gamma \in (b_i,a_{i+1})$ .

Note that the linear space ${\mathcal {W}}_i$ is the pseudo-stable space of the exponential dichotomy with any growth rate taken from the spectral gap interval $(b_i, a_{i+1})$ for $i\in \{1,\dots ,k-1\}$.

The following result on Sacker–Sell spectra of upper triangular systems follows from [9]. Note that such a statement is only true in the half-line case and does not hold for Sacker–Sell spectra on the entire time axis as demonstrated in [9].

### **Proposition 5**

(Sacker–Sell spectrum of upper triangular systems) Suppose that the linear differential equation (1) is upper triangular, i.e. $a_{ij}(t)=0$ for all $i>j$ and $t\in \mathbb {R}^+_0$, and assume that the off-diagonal elements $a_{ij}(t)$ for all $i<j$ are bounded in $t\in \mathbb {R}_0^+$. Then the Sacker–Sell spectrum of (1) coincides with that of its diagonal part $\dot{x}_i=a_{ii}(t)x_i$, $i\in \{1,\dots ,d\}$, for which, the spectrum is the union of the intervals $[\alpha _i,\beta _i]$. If also the diagonal elements of the matrix *A*(*t*) are bounded, then we have the representation

2 $$\begin{aligned} \alpha _i=\liminf _{t-s\rightarrow \infty }{1\over t-s}\int ^t_sa_{ii}(u)\,\mathrm {d}u \quad \text {and}\quad \beta _i=\limsup _{t-s\rightarrow \infty }{1\over t-s}\int ^t_sa_{ii}(u)\,\mathrm {d}u \end{aligned}$$

for all $$i\in \{1,\dots ,d\}$$.

### *Remark 6*

Note that the representation (2) does not hold if the diagonal elements of the matrix *A*(*t*) are unbounded. As a counter example consider the one-dimensional system

$$\begin{aligned} \dot{x} = a(t)x\,, \end{aligned}$$

where $a:\mathbb {R}_0^+\rightarrow \mathbb {R}$ is defined by

$$\begin{aligned} a(t) = \left\{ \begin{array}{ccl} n &{} : &{} t\in [2n,2n+1], n\in \mathbb {N}_0\,,\\ -2n-1 &{} : &{} t\in [2n+1,2n+2], n\in \mathbb {N}_0\,. \end{array} \right. \end{aligned}$$

It follows that $\int ^{n+3}_{n}a(u)\,\mathrm {d}u \le 0$ for all $n\in \mathbb {N}$, and it can be proved that

$$\begin{aligned} \lim _{t-s\rightarrow \infty }{1\over t-s} \int ^{t}_{s}a(u)\,\mathrm {d}u =-\infty \,. \end{aligned}$$

However, the Sacker–Sell spectrum is given by $[-\infty ,\infty ]$, since *a*(*t*) is arbitrarily close to $-\infty$ and $\infty$ on intervals of the length one. This shows that the representation (2) does not hold for unbounded coefficient matrices.

## The Bohl Spectrum

We first define the Bohl spectrum for each solution of (1). The Bohl spectrum of (1) is then the union over the Bohl spectra of the solutions.

### **Definition 7**

(*Bohl spectrum*) Consider the linear nonautonomous differential equation (1) in $\mathbb {R}^d$. The *Bohl spectrum of a particular solution* $X(\cdot )\xi , \xi \not =0$, of (1) is defined as

$$\begin{aligned} \Sigma _{\xi }:=\Big \{&\lambda \in \overline{\mathbb {R}}: \hbox {there exist sequences } \{t_n\}_{n\in \mathbb {N}} \hbox { and } \{s_n\}_{n\in \mathbb {N}} \\&\hbox { with } t_n-s_n\rightarrow \infty \hbox { such that } \lim _{n\rightarrow \infty } \tfrac{1}{t_n-s_n}\ln \tfrac{\Vert X(t_n)\xi \Vert }{\Vert X(s_n)\xi \Vert }=\lambda \Big \}. \end{aligned}$$

The *Bohl spectrum of* (1) is defined as

$$\begin{aligned} \Sigma _\mathrm{Bohl}:=\bigcup _{\xi \in \mathbb {R}^d\setminus \{0\}}\Sigma _{\xi }\,. \end{aligned}$$

### *Remark 8*

(i) By Definition 1, we have $\chi _{-}(\xi ), \chi _{+}(\xi )\in \Sigma _{\xi }$ for any $\xi \in \mathbb {R}^d\setminus \{0\}$, and we see that in contrast to looking at the asymptotic behavior at infinity of a solution by using the Lyapunov exponent, the Bohl spectrum of this solution provides all possible growth rates of this solution when the length of observation time tends to infinity and the initial time is arbitrary.

(ii) The *upper* and *lower Bohl exponent* of a solution $X(\cdot )\xi$ are defined by

$$\begin{aligned} \overline{\beta }(\xi ):= \limsup _{t-s\rightarrow \infty }\frac{1}{t-s}\ln \frac{\Vert X(t)\xi \Vert }{\Vert X(s)\xi \Vert } ,\quad \underline{\beta }(\xi ):= \liminf _{t-s\rightarrow \infty }\frac{1}{t-s}\ln \frac{\Vert X(t)\xi \Vert }{\Vert X(s)\xi \Vert }, \end{aligned}$$

see [14, pp. 171–172] and [5]. Thus, $\overline{\beta }(\xi )$ and $\underline{\beta }(\xi )$ measure the biggest and smallest growth rate of the solution $X(\cdot )\xi$, when the length of observation time tends to infinity, and we have

$$\begin{aligned} \overline{\beta }(\xi )=\sup \Sigma _{\xi } \quad \text {and}\quad \underline{\beta }(\xi )=\inf \Sigma _{\xi }\,. \end{aligned}$$

We note that the notion of Bohl exponent used in papers on differential algebraic equations and control theory is different (see the references cited in the Introduction).

(iii) The definition of Bohl spectrum is independent of the norm in $\mathbb {R}^d$.

(iv) A different definition of a Bohl spectrum for discrete systems depending on certain invariant splittings was proposed in [25, Definition 3.8.1], and another spectrum between the Lyapunov and Sacker–Sell spectrum based on nonuniform exponential dichotomies was introduced in [12].

Note that $\overline{\beta }(\xi )$ can be $\infty$, and $\underline{\beta }(\xi )$ can be $-\infty$. For an arbitrarily chosen $a \in \mathbb {R}$, define

$$\begin{aligned}{}[-\infty , a]:=(-\infty ,a] \cup \{-\infty \}\,,\quad \quad \quad [a, \infty ] := [a, \infty ) \cup \{\infty \} \end{aligned}$$

and

$$\begin{aligned}{}[-\infty ,-\infty ]:= \{-\infty \}, \quad \quad \ [\infty ,\infty ]:= \{\infty \}, \quad \quad \quad [-\infty ,\infty ] := \overline{\mathbb {R}}\,. \end{aligned}$$

The following proposition describes fundamental properties of the Bohl spectrum of a particular solution.

### **Proposition 9**

Consider the linear nonautonomous differential equation (1) in $\mathbb {R}^d$. For all $\xi \in \mathbb {R}^d\setminus \{0\}$, the following statements hold:

(i) We have the representation
  $$\begin{aligned} \Sigma _{\xi }:=&\Big \{\lambda \in \overline{\mathbb {R}}: \hbox { there exist sequences } \{t_n\}_{n\in \mathbb {N}}\hbox { and }\{s_n\}_{n\in \mathbb {N}}\hbox { with }\\&\quad t_n-s_n\rightarrow \infty \text { and }s_n\rightarrow \infty \hbox { such that } \lim _{n\rightarrow \infty } \tfrac{1}{t_n-s_n}\ln \tfrac{\Vert X(t_n)\xi \Vert }{\Vert X(s_n)\xi \Vert }=\lambda \Big \}\,, \end{aligned}$$
  i.e. in the definition of Bohl spectrum we can always assume $s_n\rightarrow \infty$.
(ii) $\Sigma _{\xi }=\Sigma _{\lambda \xi }$ for all $\lambda \in \mathbb {R}\setminus \{0\}$.
(iii) $$\Sigma _{\xi }=\big [\underline{\beta }(\xi ), \overline{\beta }(\xi )\big ]$$.
(iv) Suppose that there exists a constant $M>0$ such that
  3 $$\begin{aligned} \Vert A(t)\Vert \le M \quad \quad \text {for almost all }\,t\in \mathbb {R}_0^+. \end{aligned}$$
  Then $$\Sigma _{\xi } \subset [-M,M]$$.

### *Proof*

(i) Let $\lambda \in \Sigma _{\xi }$ be arbitrary. Then there exist two sequences $\{t_n\}_{n\in \mathbb {N}}$ and $\{s_n\}_{n\in \mathbb {N}}$ such that $t_n\ge s_n\ge 0$ and

4 $$\begin{aligned} \lim _{n\rightarrow \infty } t_n-s_n =\infty \quad \text {and}\quad \lim _{n\rightarrow \infty } \frac{1}{t_n-s_n} \ln \frac{\Vert X(t_n)\xi \Vert }{\Vert X(s_n)\xi \Vert }=\lambda \,. \end{aligned}$$

To conclude the proof of this part, we need to construct two sequences $\{\widetilde{t}_n\}_{n\in \mathbb {N}}$ and $\{\widetilde{s}_n\}_{n\in \mathbb {N}}$ such that

5 $$\begin{aligned} \lim _{n\rightarrow \infty }\widetilde{s}_n=\infty \,,\quad \lim _{n\rightarrow \infty }\widetilde{t}_n-\widetilde{s}_n=\infty \,, \quad \lim _{n\rightarrow \infty } \frac{1}{\widetilde{t}_n-\widetilde{s}_n} \ln \frac{\Vert X(\widetilde{t}_n)\xi \Vert }{\Vert X(\widetilde{s}_n)\xi \Vert }=\lambda \,. \end{aligned}$$

We now consider two separated cases:

*Case* 1 The sequence $\{s_n\}_{n\in \mathbb {N}}$ is unbounded. Then there exists a subsequence $\{s_{k_n}\}_{n\in \mathbb {N}}$ of $\{s_n\}_{n\in \mathbb {N}}$ such that $\lim _{n\rightarrow \infty } s_{k_n}=\infty$. Letting $\widetilde{s}_n:=s_{k_n}$ and $\widetilde{t}_n:=t_{k_n}$. Then these sequences satisfy (5).

*Case* 2 The sequence $\{s_n\}_{n\in \mathbb {N}}$ is bounded. Let $\Gamma :=\sup _{n\in \mathbb {N}} s_n$, and let $n\in \mathbb {N}$ be an arbitrary positive integer. Since $\lim _{m\rightarrow \infty } t_m-s_m=\infty$ and

$$\begin{aligned} \sup _{m\in \mathbb {N}}\left| \ln \frac{\Vert X(s_m+n)\xi \Vert }{\Vert X(s_m)\xi \Vert }\right| \le \sup _{t\in [0,\Gamma ]}\left| \ln \frac{\Vert X(t+n)\xi \Vert }{\Vert X(t)\xi \Vert }\right| <\infty \,, \end{aligned}$$

it follows that

$$\begin{aligned} \lim _{m\rightarrow \infty } \frac{1}{t_m-s_m-n}\left| \ln \frac{\Vert X(s_m+n)\xi \Vert }{\Vert X(s_m)\xi \Vert } \right| =0. \end{aligned}$$

Consequently, there exists $k_n\in \mathbb {N}$ such that

6 $$\begin{aligned} t_{k_n}-s_{k_n}\ge n^2 \quad \text {and}\quad \quad \frac{1}{t_{k_n}-s_{k_n}-n}\left| \ln \frac{\Vert X(s_{k_n}+n)\xi \Vert }{\Vert X(s_{k_n})\xi \Vert } \right| \le \frac{1}{n}. \end{aligned}$$

Define two sequences $\{\widetilde{t}_n\}_{n\in \mathbb {N}}$ and $\{\widetilde{s}_n\}_{n\in \mathbb {N}}$ by

$$\begin{aligned} \widetilde{t}_n=t_{k_n} \quad \text {and}\quad \widetilde{s}_n:=s_{k_n}+n \quad \text {for all }\,n\in \mathbb {N}\,, \end{aligned}$$

where $k_n$ satisfies (6). Obviously, $\lim _{n\rightarrow \infty }\widetilde{s}_n=\infty , \lim _{n\rightarrow \infty }\widetilde{t}_n-\widetilde{s}_n=\infty$. It remains to compute $\lim _{n\rightarrow \infty }\frac{1}{\widetilde{t}_n-\widetilde{s}_n}\ln \frac{\Vert X(\widetilde{t}_n)\xi \Vert }{\Vert X(\widetilde{s}_n)\xi \Vert }$. By definition of $\{\widetilde{t}_n\}_{n\in \mathbb {N}}$ and $\{\widetilde{s}_n\}_{n\in \mathbb {N}}$, we have

$$\begin{aligned} \frac{1}{\widetilde{t}_n-\widetilde{s}_n}\ln \frac{\Vert X(\widetilde{t}_n)\xi \Vert }{\Vert X(\widetilde{s}_n)\xi \Vert }&= \frac{1}{t_{k_n}-s_{k_n}-n}\ln \frac{\Vert X(t_{k_n})\xi \Vert }{\Vert X(s_{k_n}+n)\xi \Vert }\\&= \frac{1}{t_{k_n}-s_{k_n}-n} \left( \ln \frac{\Vert X(t_{k_n})\xi \Vert }{\Vert X(s_{k_n})\xi \Vert }+\ln \frac{\Vert X(s_{k_n})\xi \Vert }{\Vert X(s_{k_n}+n)\xi \Vert }\right) . \end{aligned}$$

Using (6), we obtain that

7 $$\begin{aligned} \left| \frac{1}{\widetilde{t}_n-\widetilde{s}_n}\ln \frac{\Vert X(\widetilde{t}_n)\xi \Vert }{\Vert X(\widetilde{s}_n)\xi \Vert }- \frac{1}{t_{k_n}-s_{k_n}-n} \ln \frac{\Vert X(t_{k_n})\xi \Vert }{\Vert X(s_{k_n})\xi \Vert } \right| \le \frac{1}{n}. \end{aligned}$$

On the other hand, from $t_{k_n}-s_{k_n}\ge n^2$, we derive that $\lim _{n\rightarrow \infty }\frac{t_{k_n}-s_{k_n}}{t_{k_n}-s_{k_n}-n}=1$ and therefore

$$\begin{aligned} \lim _{n\rightarrow \infty } \frac{1}{t_{k_n}-s_{k_n}-n} \ln \frac{\Vert X(t_{k_n})\xi \Vert }{\Vert X(s_{k_n})\xi \Vert } = \lim _{n\rightarrow \infty } \frac{1}{t_{k_n}-s_{k_n}} \ln \frac{\Vert X(t_{k_n})\xi \Vert }{\Vert X(s_{k_n})\xi \Vert } =\lambda , \end{aligned}$$

which together with (7) implies that the sequences $\{\widetilde{t}_n\}_{n\in \mathbb {N}}$ and $\{\widetilde{s}_n\}_{n\in \mathbb {N}}$ satisfy (5) and the proof of this part is complete.

(ii) This assertion follows directly from Definition 7.

(iii) Let $a<b$ be in $\Sigma _{\xi }$, and choose $\lambda \in (a,b)$ arbitrarily. Then there exist sequences $\{t_n\}_{n\in \mathbb {N}}, \{s_n\}_{n\in \mathbb {N}}$, $\{\tau _n\}_{n\in \mathbb {N}}$ and $\{\sigma _n\}_{n\in \mathbb {N}}$ such that $t_n-s_n > n$, $\tau _n-\sigma _n>n$,

$$\begin{aligned} \lim _{n\rightarrow \infty }\frac{1}{t_n-s_n} \ln \frac{\Vert X(t_n)\xi \Vert }{\Vert X(s_n)\xi \Vert }=a \quad \text {and}\quad \lim _{n\rightarrow \infty }\frac{1}{\tau _n-\sigma _n} \ln \frac{\Vert X(\tau _n)\xi \Vert }{\Vert X(\sigma _n)\xi \Vert }=b\,. \end{aligned}$$

Consequently, there exists $N\in \mathbb {N}$ such that for all $n\ge N$,

8 $$\begin{aligned} \frac{1}{t_n-s_n} \ln \frac{\Vert X(t_n)\xi \Vert }{\Vert X(s_n)\xi \Vert } <\lambda < \frac{1}{\tau _n-\sigma _n} \ln \frac{\Vert X(\tau _n)\xi \Vert }{\Vert X(\sigma _n)\xi \Vert }\,. \end{aligned}$$

Consider the following continuous function $g:[0,1]\rightarrow \mathbb {R}$ defined by

$$\begin{aligned} g(\theta ):=\frac{1}{ \theta (t_n-s_n)+(1-\theta )(\tau _n-\sigma _n)}\ln \frac{\Vert X(\theta t_n+(1-\theta )\tau _n)\xi \Vert }{\Vert X(\theta s_n+(1-\theta )\sigma _n)\xi \Vert }\,. \end{aligned}$$

From (8), we have $g(0)>\lambda >g(1)$, and by the Intermediate Value Theorem, there exists $\theta _n\in (0,1)$ such that $g(\theta _n)=\lambda$. This together with the fact that $\lim _{n\rightarrow \infty } \theta _n(t_n-s_n)+(1-\theta _n)(\tau _n-\sigma _n)=\infty$ implies that $\lambda \in \Sigma _{\xi }$ and completes the proof.

(iv) Let $\xi \in \mathbb {R}^d\setminus \{0\}$ be arbitrary. We have the integral equality

$$\begin{aligned} X(t)\xi =X(s)\xi +\int _s^{t} A(u)X(u)\xi \,\mathrm {d}u \quad \text {for all }\,t\ge s\,. \end{aligned}$$

Thus,

$$\begin{aligned} \Vert X(t)\xi \Vert \le \Vert X(s)\xi \Vert +M\int _s^t \Vert X(u)\xi \Vert \,\mathrm {d}u\,. \end{aligned}$$

Applying Gronwall’s inequality yields that

$$\begin{aligned} \Vert X(t)\xi \Vert \le e^{M(t-s)}\Vert X(s)\xi \Vert \quad \text {for all }\,t\ge s\ge 0\,, \end{aligned}$$

which implies that $\sup \Sigma _{\xi }\le M$. Similarly, $\inf \Sigma _{\xi }\ge -M$. Hence, by (iii), $\Sigma _{\xi }=[\inf \Sigma _{\xi },\sup \Sigma _{\xi }]\subset [-M,M]$, which completes the proof.$\square$

### **Proposition 10**

Consider a linear nonautonomous differential equation $\dot{x}=A(t)x$ in $\mathbb {R}^d$, and let *x*(*t*), *y*(*t*) be solutions such that the angle between them is bounded below by a positive number. Then if $\alpha \beta \ne 0$, the solutions $t\mapsto \alpha x(t)+\beta y(t)$ all have the same Bohl spectrum.

### *Proof*

We use the Euclidean norm $\Vert \cdot \Vert$ on $\mathbb {R}^d$. Without loss of generality, we may assume that $\alpha =1$. So we consider the solutions

$$\begin{aligned} z(t)=x(t)+\beta y(t). \end{aligned}$$

If we define

$$\begin{aligned} e_1(t)=\frac{x(t)}{\Vert x(t)\Vert } \quad \text {and}\quad e_2(t)=\frac{y(t)}{\Vert y(t)\Vert }\,, \end{aligned}$$

we see that

$$\begin{aligned} z(t)=\Vert x(t)\Vert e_1(t)+\beta \Vert y(t)\Vert e_2(t) \end{aligned}$$

and

$$\begin{aligned} \Vert x(t)\Vert = (1-\langle e_1(t),e_2(t)\rangle ^2)^{-1}[\langle z(t),e_1(t)\rangle -\langle e_1(t),e_2(t)\rangle \cdot \langle z(t),e_2(t)\rangle ] \end{aligned}$$

and

$$\begin{aligned} \beta \Vert y(t)\Vert = (1-\langle e_1(t),e_2(t)\rangle ^2)^{-1}[-\langle e_1(t),e_2(t)\rangle \cdot \langle z(t),e_1(t)\rangle +\langle z(t),e_2(t)\rangle ]\,. \end{aligned}$$

By the angle assumption, we have $1-\langle e_1(t),e_2(t)\rangle ^2\ge \delta$ for some $\delta >0$. This implies

$$\begin{aligned} \Vert z(t)\Vert \le 2\max \{\Vert x(t)\Vert , |\beta | \Vert y(t)\Vert \} \le \frac{4}{\delta }\Vert z(t)\Vert \,. \end{aligned}$$

Now let $z_1(t)$ correspond to $\beta _1$ and $z_2(t)$ to $\beta _2$. Then we note that

$$\begin{aligned} \frac{\Vert z_1(t)\Vert }{\Vert z_2(t)\Vert } \le \frac{4}{\delta }\frac{\max \{\Vert x(t)\Vert , |\beta _1| \Vert y(t)\Vert \}}{\max \{\Vert x(t)\Vert , |\beta _2| \Vert y(t)\Vert \}} \le \frac{4R}{\delta r}\,, \end{aligned}$$

where $R=\max \{|\beta _1|,|\beta _2|\}$ and $r=\min \{|\beta _1|,|\beta _2|\}$. Of course, we can interchange the indices 1 and 2 here. Then

$$\begin{aligned} \frac{\Vert z_1(t)\Vert }{\Vert z_1(s)\Vert } = \frac{\Vert z_1(t)\Vert }{\Vert z_2(t)\Vert } \frac{\Vert z_2(t)\Vert }{\Vert z_2(s)\Vert } \frac{\Vert z_2(s)\Vert }{\Vert z_1(s)\Vert } \le \frac{16R^2}{\delta ^2 r^2}\frac{\Vert z_2(t)\Vert }{\Vert z_2(s)\Vert }=N\frac{\Vert z_2(t)\Vert }{\Vert z_2(s)\Vert }\,, \end{aligned}$$

where $N:= \frac{16R^2}{\delta ^2 r^2}$. It follows that

$$\begin{aligned} \frac{1}{t-s}\ln \frac{\Vert z_1(t)\Vert }{\Vert z_1(s)\Vert }\le \frac{\ln \,N}{t-s}+ \frac{1}{t-s}\ln \frac{\Vert z_2(t)\Vert }{\Vert z_2(s)\Vert }\,. \end{aligned}$$

Thus,

$$\begin{aligned} \limsup _{t-s\rightarrow \infty }\frac{1}{t-s}\ln \frac{\Vert z_1(t)\Vert }{\Vert z_1(s)\Vert }\le \limsup _{t-s\rightarrow \infty }\frac{1}{t-s}\ln \frac{\Vert z_2(t)\Vert }{\Vert z_2(s)\Vert }\,. \end{aligned}$$

Switching the indices 1 and 2, we get equality. Next from

$$\begin{aligned} \frac{1}{t-s}\ln \frac{\Vert z_2(t)\Vert }{\Vert z_2(s)\Vert }\ge -\frac{\ln \,N}{t-s}+ \frac{1}{t-s}\ln \frac{\Vert z_1(t)\Vert }{\Vert z_1(s)\Vert }\,, \end{aligned}$$

we get

$$\begin{aligned} \liminf _{t-s\rightarrow \infty }\frac{1}{t-s}\ln \frac{\Vert z_2(t)\Vert }{\Vert z_2(s)\Vert }\ge \liminf _{t-s\rightarrow \infty }\frac{1}{t-s}\ln \frac{\Vert z_1(t)\Vert }{\Vert z_1(s)\Vert } \end{aligned}$$

and switching the indices 1 and 2, we get equality also. The conclusion is that $z_1(t)$ and $z_2(t)$ have the same Bohl spectrum. $\square$

### *Remark 11*

We demonstrate that the common Bohl spectrum of the solution $t\mapsto \alpha x(t)+\beta y(t)$ in Proposition 10 does not depend just on $\Sigma _x$ and $\Sigma _y$. Consider the diagonal system

$$\begin{aligned} \dot{x}&=0\,,\\ \dot{y}&=a(t)y\,, \end{aligned}$$

where $a(t)=1$ if $T_{2k}\le t\le T_{2k+1}$, and $a(t)=-1$ if $T_{2k+1}\le t\le T_{2k+2}$. Here $T_k$ is an increasing sequence with $T_0=0$ and $T_{k+1}-T_k\rightarrow \infty$ as $k\rightarrow \infty$. Then if we take the solutions $x(t)=(1,0)$ and $y(t)=\big (0,\exp (\int ^t_0a(u)\,\mathrm {d}u)\big )$, it is easy to see that $\Sigma _x=\{0\}$ and $\Sigma _y=[-1,1]$. By appropriate choice of the sequence $T_k$, we can arrange that $\int ^t_0a(u)\,\mathrm {d}u\ge 0$ for $t\ge 0$. Then if we use the maximum norm in $\mathbb {R}^2$, we see that $\Vert x(t)+y(t)\Vert =|y(t)|$ for all $t\ge 0$. This means that for all $t\ge s\ge 0$, we have

$$\begin{aligned} \frac{1}{t-s}\ln \frac{\Vert x(t)+y(t)\Vert }{\Vert x(s)+y(s)\Vert }=\frac{1}{t-s}\ln \frac{\Vert y(t)\Vert }{\Vert y(s)\Vert }. \end{aligned}$$

It follows that $\Sigma _{x+y}=\Sigma _y$.

On the other hand, again by appropriate choice of the sequence $T_k$, we can arrange that $\int ^t_0a(u)\,\mathrm {d}u\le 0$ for $t\ge T_2$. Then if we use the maximum norm in $\mathbb {R}^2$, we see that $\Vert x(t)+y(t)\Vert =|x(t)|=1$ for all $t\ge T_2$. So for all $t\ge T_2$ and $s\ge T_2$, we get

$$\begin{aligned} \frac{1}{t-s}\ln \frac{\Vert x(t)+y(t)\Vert }{\Vert x(s)+y(s)\Vert }=\frac{1}{t-s}\ln \frac{1}{1}=0\,, \end{aligned}$$

which implies that $\Sigma _{x+y}=\Sigma _x$.

## Spectral Theorem

We prove in this section that the Bohl spectrum of a locally integrable linear nonautonomous differential equation consists of at most finitely many intervals, the number of which is bounded by the dimension of the system, and we associate a filtration of subspaces to these spectral intervals.

### **Theorem 12**

(Bohl Spectral Theorem) Consider the linear nonautonomous differential equation (1) in $\mathbb {R}^d$. Then its Bohl spectrum consists of *k* (not necessarily closed) disjoint intervals, i.e.

$$\begin{aligned} \Sigma _\mathrm{Bohl}=I_1\cup \dots \cup I_k, \end{aligned}$$

where $1\le k\le d$ and $I_1,I_2,\ldots ,I_k$ are ordered intervals. The interval $I_1$ can be unbounded from below, $I_k$ can be unbounded from above, and $I_2,\dots ,I_{k-1}$ are bounded. There exists a corresponding filtration

9 $$\begin{aligned} \{0\}={\mathcal {S}}_0 \subsetneq {\mathcal {S}}_1\subsetneq {\mathcal {S}}_2\subsetneq \dots \subsetneq \mathcal {S}_k=\mathbb {R}^d \end{aligned}$$

satisfying the following dynamical characterization

10 $$\begin{aligned} {\mathcal {S}}_i\setminus \{0\}= \big \{\xi \in \mathbb {R}^d: \Sigma _{\xi }\subset \textstyle \bigcup _{j=1}^i I_j \big \}\quad \text {for all }\,i\in \{1,\ldots ,k\}\,. \end{aligned}$$

### *Proof*

Let $\lambda \in \overline{\mathbb {R}}\setminus \Sigma _\mathrm{Bohl}$ be arbitrary. Due to Proposition 9 (iii), for any $\xi \in \mathbb {R}^d\setminus \{0\}$, either $\Sigma _{\xi }\subset [-\infty ,\lambda )$ or $\Sigma _{\xi }\subset (\lambda ,+\infty ]$ holds. Define

11 $$\begin{aligned} {\mathcal {M}}_{\lambda }:= \big \{ \xi \in \mathbb {R}^d\setminus \{0\}: \Sigma _{\xi }\subset [-\infty ,\lambda ) \big \}\cup \{0\} \end{aligned}$$

and

$$\begin{aligned} {\mathcal {N}}_{\lambda }:= \big \{ \xi \in \mathbb {R}^d\setminus \{0\}: \Sigma _{\xi }\subset (\lambda ,\infty ] \big \}\,. \end{aligned}$$

Obviously, ${\mathcal {M}}_{\lambda }\cup {\mathcal {N}}_{\lambda }=\mathbb {R}^d$, and we show now that ${\mathcal {M}}_{\lambda }$ is a linear subspace of $\mathbb {R}^d$. Consider $\xi ,\eta \in {\mathcal {M}}_{\lambda }$ and $\alpha ,\beta \in \mathbb {R}$ with $\alpha \xi +\beta \eta \not =0$. Since $\Sigma _{\xi },\Sigma _{\eta }\subset [-\infty ,\lambda )$, it follows that

$$\begin{aligned} \limsup _{t\rightarrow \infty }\frac{1}{t}\ln \Vert X(t)\xi \Vert <\lambda \quad \text {and}\quad \limsup _{t\rightarrow \infty }\frac{1}{t}\ln \Vert X(t)\eta \Vert <\lambda \,, \end{aligned}$$

which implies that there exist $K>0$ and $\mu <\lambda$ such that

$$\begin{aligned} \max \{\Vert X(t)\xi \Vert , \Vert X(t)\eta \Vert \} \le K e^{\mu t}\quad \text {for all }\,t\ge 0\,. \end{aligned}$$

Consequently,

$$\begin{aligned} \Vert X(t)(\alpha \xi +\beta \eta )\Vert \le K (|\alpha |+|\beta |) e^{\mu t}\quad \text {for all }\,t\ge 0\,. \end{aligned}$$

Hence, there exists a sequence $\{t_n\}_{n\in \mathbb {N}}$ tending to infinity with

$$\begin{aligned} \lim _{n\rightarrow \infty }\frac{1}{t_n}\ln \Vert X(t_n)(\alpha \xi +\beta \eta )\Vert \in [-\infty ,\lambda )\,. \end{aligned}$$

Thus, $\Sigma _{\alpha \xi +\beta \eta }\cap [-\infty ,\lambda )\not =\emptyset$, and since $\Sigma _{\alpha \xi +\beta \eta }$ is an interval that does not contain $\lambda$, it must be a subset of $[-\infty ,\lambda )$, and thus, we have $\alpha \xi +\beta \eta \in {\mathcal {M}}_{\lambda }$. Hence, ${\mathcal {M}}_{\lambda }$ is a linear subspace of $\mathbb {R}^d$.

Let $d_0<d_1<\dots <d_n$ be elements of the set $\{\dim ({\mathcal {M}}_{\lambda }): \lambda \in \overline{\mathbb {R}}\setminus \Sigma _\mathrm{Bohl}\}$. Depending on whether $\pm \infty \in \Sigma _\mathrm{Bohl}$ or not, we have the following estimate on the number *n*:

- If $\pm \infty \in \Sigma _\mathrm{Bohl}$, then $d_0\ge 1$ and $d_n\le d-1$ and therefore $n\le d-2$.
- If $-\infty \in \Sigma _\mathrm{Bohl}$ and $+\infty \not \in \Sigma _\mathrm{Bohl}$, then $d_0\ge 1$ and therefore $n\le d-1$.
- If $-\infty \not \in \Sigma _\mathrm{Bohl}$ and $+\infty \in \Sigma _\mathrm{Bohl}$, then $d_n\le d-1$ and therefore $n\le d-1$.
- If $-\infty \not \in \Sigma _\mathrm{Bohl}$ and $+\infty \not \in \Sigma _\mathrm{Bohl}$, then $n\le d$.

For $i\in \{0,\dots ,n\}$, we define

12 $$\begin{aligned} J_i:=\big \{\lambda \in \overline{\mathbb {R}}\setminus \Sigma _\mathrm{Bohl}: \dim ({\mathcal {M}}_{\lambda })=d_i\big \}. \end{aligned}$$

We now show that each set $J_i$ is an interval. Let $i\in \{0,\dots ,n\}$ and $a<b$ be two elements of $J_i$. We show now that $[a,b]\subset J_i$. By (11), we have ${\mathcal {M}}_{a}\subset {\mathcal {M}}_{b}$, and using $\dim ({\mathcal {M}}_{a})=\dim ({\mathcal {M}}_{b})$, this implies that ${\mathcal {M}}_{a}={\mathcal {M}}_{b}$, and thus ${\mathcal {N}}_{a}={\mathcal {N}}_{b}$. This means that $\mathbb {R}^d={\mathcal {M}}_{a}\cup {\mathcal {N}}_b$. Let $\lambda \in [a,b]$ be arbitrary and $\xi \in \mathbb {R}^d\setminus \{0\}$. Thus, either $\xi \in {\mathcal {M}}_{a}$ or $\xi \in {\mathcal {N}}_b$. In both of these cases, we have $\lambda \not \in \Sigma _{\xi }$ and therefore $\lambda \in \overline{\mathbb {R}}\setminus \Sigma _\mathrm{Bohl}$. Now, we know that ${\mathcal {M}}_{\lambda }$ is a linear subspace and by (11) we have ${\mathcal {M}}_{a}\subset {\mathcal {M}}_{\lambda }\subset {\mathcal {M}}_{b}$. Thus, ${\mathcal {M}}_{a}={\mathcal {M}}_{\lambda }={\mathcal {M}}_{b}$ and therefore $\lambda \in J_i$. This means that we have proved that $J_i$ is an interval. Obviously, the order of the intervals $J_0,\dots ,J_n$ is $J_0<J_1<\dots <J_n$ and we have

$$\begin{aligned} \Sigma _\mathrm{Bohl}=\overline{\mathbb {R}}\setminus \bigcup _{i=0}^n J_i. \end{aligned}$$

Let *k* denote the number of disjoint intervals $I_i$ of $\Sigma _\mathrm{Bohl}$. According to the cases (a–d) above, we have the following dependence of *k* and *n*:

(i) $k=n+2$ in case (a) above,
(ii) $k=n+1$ in case (b) and (c) above,
(iii) $k=n$ in case (d) above.

Thus, from the relation between *n* and *d* established above, we always obtain that $k\le d$. To conclude the proof, for each $i\in \{1,\dots ,k\}$, we define the set ${\mathcal {S}}_i$ as in (10) together with $\{0\}$. Note that the space $\mathcal {S}_i$ coincides with $\mathcal {M}_\lambda$ for $\lambda = \frac{1}{2} (\sup I_i + \inf I_{i+1})$, where $i\in \{1,\dots ,k-1\}$, and $\mathcal {S}_k=\mathcal {M}_\lambda =\mathbb {R}^d$ for $\lambda > \sup I_k$. Then, clearly ${\mathcal {S}}_i$ is a linear subspace and satisfies (9). This finishes the proof. $\square$

Next, we concentrate on constructing an example of a nonautonomous differential equation such that its Bohl spectrum is not closed. Our construction is implicit by using a result from [4]:

Let ${\mathcal {M}}_d$ denote the set of all piecewise continuous and uniformly bounded matrix-valued functions $A:\mathbb {R}_0^+\rightarrow \mathbb {R}^{d\times d}$. For each $A\in {\mathcal {M}}_d$, consider the linear nonautonomous differential equation

13 $$\begin{aligned} \dot{x}=A(t)x\,. \end{aligned}$$

Consider the *uniform upper exponent function* of (13), $\overline{\beta }_A:\mathbb {R}^{d}\setminus \{0\}\rightarrow \mathbb {R}$, where $\overline{\beta }_A(\xi )$ is the upper Bohl exponent of the solution $X(t)\xi$ of (13). A complete description of the set of functions $\overline{{\mathcal {B}}}_d:=\{\overline{\beta }_A: A\in {\mathcal {M}}_d\}$ is given as follows (see [4, Theorem 1]).

### **Theorem 13**

A function $\beta :\mathbb {R}^d\setminus \{0\}\rightarrow \mathbb {R}$ belongs to the class $\overline{{\mathcal {B}}}_d$ if and only if it satisfies the following three conditions:

(i) $$\beta $$ is bounded.
(ii) $\beta (\xi )=\beta (\alpha \xi )$ for any nonzero $\alpha \in \mathbb {R}$ and $\xi \in \mathbb {R}^d\setminus \{0\}$.
(iii) For any $q\in \mathbb {R}$, the set $\{\xi : \beta (\xi )\ge q\}$ is a $G_{\delta }$ set.

The following example shows that the intervals of the Bohl spectrum do not need to be closed.

### *Example 14*

Consider the function $\beta :\mathbb {R}^2\setminus \{0\}\rightarrow \mathbb {R}$ defined by

14 $$\begin{aligned} \beta (r\cos \varphi ,r\sin \varphi )= \left\{ \begin{array}{ll} 0 &{} : \varphi =0, \\ |\cos \varphi |&{} : \varphi \in (0,2\pi ), \end{array} \right. \end{aligned}$$

where $r\in (0,\infty )$. Obviously, the function $\beta$ satisfies the three conditions of Theorem 13. Consequently, there exists a piecewise continuous and uniformly bounded matrix-valued function $A:\mathbb {R}_0^+\rightarrow \mathbb {R}^{2\times 2}$ such that $\overline{\beta }_A\equiv \beta$. By construction of $\beta _A$, it is easy to see that $[0,1)\subset \Sigma _\mathrm{Bohl}$. Suppose to the contrary that $\Sigma _\mathrm{Bohl}$ is closed. Thus, $1\in \Sigma _\mathrm{Bohl}$, which means there exists $\xi \in \mathbb {R}^2\setminus \{0\}$ such that $1\in \Sigma _{\xi }$. That leads to a contradiction, since $\overline{\beta }_A(\xi )<1$. Thus, $\Sigma _\mathrm{Bohl}$ is not closed.

In the remaining part of this section, we show that Bohl spectrum is preserved under a kinematic similarity transformation. Recall that a linear nonautonomous differential equation

15 $$\begin{aligned} \dot{x}=A(t)x \quad \text {for all }\,t\in \mathbb {R}_0^+ \end{aligned}$$

is said to be *kinematically similar* to another linear nonautonomous differential equation

16 $$\begin{aligned} \dot{y}=B(t)y \quad \text {for all }\,t\in \mathbb {R}_0^+ \end{aligned}$$

if there exists a continuously differentiable function $S:\mathbb {R}^+_0\rightarrow \mathbb {R}^{d\times d}$ of invertible matrices such that both *S* and $S^{-1}$ are bounded, and which satisfies the differential equation

17 $$\begin{aligned} \dot{S}(t)=A(t)S(t)-S(t)B(t) \quad \text {for all }\,t\in \mathbb {R}_0^+ \end{aligned}$$

(see [13, p. 38]).

### **Proposition 15**

(Invariance of the Bohl spectrum under kinematic similarity transformations) Suppose that (15) and (16) are kinematically similar. Then the Bohl spectra $\Sigma _\mathrm{Bohl}(A)$ and $\Sigma _\mathrm{Bohl}(B)$ of (15) and (16) coincide.

### *Proof*

Let $X_A(t)$ and $X_B(t)$ denote the fundamental matrix solution of (15) and (16), respectively. From (17), we derive

$$\begin{aligned} X_B(t)=S(t)^{-1}X_A(t)S(0) \quad \text {for all }\,t\ge 0\,, \end{aligned}$$

which implies that the Bohl spectrum of the solution $X_A(t)S(0)\xi$ of (15) is equal to the Bohl spectrum of the solution $X_B(t)\xi$ for all $\xi \in \mathbb {R}^d\setminus \{0\}$, where we use the inequality $\ln \Vert y \Vert -\ln \Vert S^{-1}(t)\Vert \le \ln \Vert S(t)y\Vert \le \ln \Vert y\Vert +\ln \Vert S(t)\Vert$. Since *S*(0) is invertible it follows that $\Sigma _\mathrm{Bohl}(A)=\Sigma _\mathrm{Bohl}(B)$ and the proof is complete. $\square$

## Bohl and Sacker–Sell Spectrum

This section is devoted to the comparison of the Bohl spectrum with the Sacker–Sell spectrum. Note that the one-dimensional example discussed in Remark 6 is an unbounded system for which the both spectra do not coincide, since the Bohl spectrum of this differential equation is given by $\{-\infty \}$. It follows also directly from Example 14 that the Bohl spectrum does not always coincide with the Sacker–Sell spectrum, since the Sacker–Sell spectrum consists of closed intervals. In this section, we give an explicit example of a bounded two-dimensional system for which the Sacker–Sell spectrum is a nontrivial interval and the Bohl spectrum is a single point. We also show that the Bohl spectrum is always a subset of the Sacker–Sell spectrum, and we provide sufficient conditions under which both spectra coincide.

### The Bohl Spectrum Can Consist of One Point, When the Sacker–Sell Spectrum is a Non-trivial Interval

Consider a $\delta > 0$ and an increasing sequence of non-negative numbers $\{T_k\}_{k\in \mathbb {N}_0}$ satisfying $T_0=0$ and the conditions

18 $$\begin{aligned} \lim _{k\rightarrow \infty } (T_{k+1}-T_k)=\infty \quad \text {and}\quad \lim _{k\rightarrow \infty } \frac{e^{T_{2k+2}-T_{2k+1}}}{T_{2k+1}-T_{2k}}=0\,. \end{aligned}$$

An example of such a sequence $\{T_k\}_{k\in \mathbb {N}}$ is $T_0=0$ and

$$\begin{aligned} T_{k+1} := \left\{ \begin{array}{ccl} T_k+ e^{k^2}&{} : &{} k \hbox { is even}\,, \\ T_k+ k &{} : &{} \hbox {otherwise}\,. \end{array} \right. \end{aligned}$$

Define a piecewise constant matrix-valued function $A:\mathbb {R}_0^+\rightarrow \mathbb {R}^{2\times 2}$ by

19 $$\begin{aligned} A(t):=\left\{ \begin{array}{ccl} A_1 &{} : &{} T_{2k}\le t\le T_{2k+1}\,, \\ A_2 &{} : &{} T_{2k+1}\le t\le T_{2k+2}\,, \end{array}\right. \end{aligned}$$

where

$$\begin{aligned} A_1:=\left( \begin{matrix} -1&{} \delta \\ 0&{}-1\end{matrix}\right) \quad \text {and}\quad A_2:=\left( \begin{matrix} -1&{} 0\\ 0&{}0\end{matrix}\right) \,. \end{aligned}$$

#### **Proposition 16**

Consider the bounded system

20 $$\begin{aligned} \dot{x}=A(t)x, \end{aligned}$$

where $A:\mathbb {R}_0^+\rightarrow \mathbb {R}^{2\times 2}$ is defined as in (19). Then the Bohl spectrum $\Sigma _\mathrm{Bohl}$ and the Sacker–Sell spectrum $\Sigma _\mathrm{SS}$ of this system are given by

$$\begin{aligned} \Sigma _\mathrm{Bohl}=\{-1\} \quad \text {and}\quad \Sigma _\mathrm{SS}=[-1,0]\,, \end{aligned}$$

respectively.

Before proving the above proposition, we need the following lemma.

#### **Lemma 17**

Let $t\mapsto (x(t),y(t))$ be an arbitrary nonzero solution of (20) with $y(0)\not =0$. Then there exists $T>0$ such that *x*(*t*) and *y*(*t*) have the same sign for all $t\ge T$ .

#### *Proof*

The flows for the autonomous systems $\dot{x}=A_1x$ and $\dot{x}=A_2x$ are given by

$$\begin{aligned} e^{A_1 t}=e^{-t}\left( \begin{matrix} 1&{} \delta t\\ 0&{}1\end{matrix}\right) \quad \text {and}\quad e^{A_2 t}=\left( \begin{matrix} e^{-t}&{} 0\\ 0&{}1\end{matrix}\right) \,, \end{aligned}$$

respectively. First suppose that $y(0)>0$, and without loss of generality assume that $y(0)=1$. We show by induction that

21 $$\begin{aligned} x(T_{2k+1}) \ge e^{-T_{2k+1}}\left( x(0)+\delta \sum _{\ell =0}^k (T_{2\ell +1}- T_{2\ell })\right) \quad \text {for all }\,k\in \mathbb {N}_0\,. \end{aligned}$$

This is clearly true for $k=0$, since we have $x(T_1)= e^{-T_1}\big (x(0)+\delta T_1\big )$. We now assume that (21) is true for a fixed $k\in \mathbb {N}_0$, and we prove (21) for $k+1$. This follows from

$$\begin{aligned} x(T_{2k+3})&= e^{-(T_{2k+3}-T_{2k+2})}\big (x(T_{2k+2})+\delta (T_{2k+3}-T_{2k+2})y(T_{2k+2})\big )\\&= e^{-(T_{2k+3}-T_{2k+1})}x(T_{2k+1}) +e^{-(T_{2k+3}-T_{2k+2})}\delta (T_{2k+3}-T_{2k+2})y(T_{2k+2})\\&\ge e^{-T_{2k+3}}\Big (\big (x(0)+\delta \textstyle \sum _{\ell =0}^k (T_{2\ell +1}- T_{2\ell })\big ) \\&\quad +e^{T_{2k+2}}\delta (T_{2k+3}-T_{2k+2})y(T_{2k+2})\Big )\\&\ge e^{-T_{2k+3}}\big (x(0)+\delta \textstyle \sum _{\ell =0}^{k+1} (T_{2\ell +1}- T_{2\ell })\big )\,, \end{aligned}$$

where the last inequality follows from $e^{-t} y(t) \ge 1$ for all $t\ge 0$.

It follows from (21) that there exists a $k\in \mathbb {N}$ with $x(T_{2k+1})\ge 0$, and we also have $y(T_{2k+1})>0$. Since the first quadrant is positively invariant under both systems, it follows that $x(t)\ge 0$ and $y(t)\ge 0$ for $t\ge T_{2k+1}$. If $y(0)<0$, we get the required result by considering the solution $-(x(t),y(t))$. $\square$

#### *Proof of Proposition 16*

We first show that $\Sigma _\mathrm{SS}=[0,1]$. Due to Proposition 5, the Sacker–Sell spectrum of this upper triangular system coincides with that for the diagonal system diag(*a*(*t*), *b*(*t*)), where $a(t) = -1$ and $-1 \le b(t) \le 0$. $\dot{x}=a(t)x$ has Sacker–Sell spectrum $\{-1\}$, and the Sacker–Sell spectrum of $\dot{y}=b(t)y$ is contained in $[-1,0]$. However,

$$\begin{aligned}\frac{1}{T_{2k}-T_{2k-1}}\int ^{T_{2k}}_{T_{2k-1}}b(t)\,\mathrm {d}t \rightarrow 0\quad \mathrm{and}\quad \frac{1}{T_{2k+1}-T_{2k}}\int ^{T_{2k+1}}_{T_{2k}}b(t)\,\mathrm {d}t \rightarrow -1\,,\end{aligned}$$

so the Sacker–Sell spectrum of $\dot{y}=b(t)y$ is exactly $[-1,0]$, and thus, the Sacker–Sell spectrum of the whole system is $[-1,0]$.

We now show that $\Sigma _\mathrm{Bohl}=\{-1\}$ by proving that for fixed $\xi \in \mathbb {R}^2\setminus \{0\}$, we have $\Sigma _{\xi }=\{-1\}$. For this purpose, write $(x(t),y(t))=X(t)\xi$ and let $\varepsilon \in (0,\delta )$ be arbitrary. By (18) and Lemma 17, assuming without loss of generality that $y(0)\ne 0$, there exists a $K\in \mathbb {N}$ such that *x*(*t*) and *y*(*t*) have the same sign for $t\ge T_{2K}$ and

22 $$\begin{aligned} \frac{e^{T_{2k+2}-T_{2k+1}}}{T_{2k+1}-T_{2k}}\le \varepsilon \quad \text {for all }\,k\ge K\,. \end{aligned}$$

We show that

23 $$\begin{aligned} \frac{|x(t)|}{|y(t)|} \ge \frac{\delta }{\varepsilon } >1 \quad \text {for all }\,t\ge T_{2K+2}\,. \end{aligned}$$

Firstly, since

$$\begin{aligned} X(T_{2k+1})X^{-1}(T_{2k}) =e^{-(T_{2k+1}-T_{2k})} \left( \begin{matrix} 1 &{} \delta (T_{2k+1}-T_{2k})\\ 0 &{} 1 \end{matrix}\right) \quad \text {for all }\,k\ge K\,, \end{aligned}$$

it follows that

24 $$\begin{aligned} \frac{|x(T_{2k+1})|}{|y(T_{2k+1})|} =\frac{|x(T_{2k})+\delta (T_{2k+1}-T_{2k})y(T_{2k})|}{|y(T_{2k})|} \ge \delta (T_{2k+1}-T_{2k})\,. \end{aligned}$$

Then for $t\in [T_{2k+2},T_{2k+3}]$ and $k\ge K$, we have

$$\begin{aligned} \frac{|x(t)|}{|y(t)|}&= \frac{|x(T_{2k+2})+\delta (t-T_{2k+2})y(T_{2k+2})|}{|y(T_{2k+2})|} \ge \frac{|x(T_{2k+2})|}{|y(T_{2k+2})|}\\&= e^{-(T_{2k+2}-T_{2k+1})} \frac{|x(T_{2k+1})|}{|y(T_{2k+1})|}\mathop {\ge }\limits ^{(24)} e^{-(T_{2k+2}-T_{2k+1})}\delta (T_{2k+1}-T_{2k})\mathop {\ge }\limits ^{(22)} \frac{\delta }{\varepsilon }\,. \end{aligned}$$

Next for $t\in [T_{2k+3},T_{2k+4}]$ and $k\ge K$, we have, using the inequality (24), that

$$\begin{aligned} \frac{|x(t)|}{|y(t)|} = e^{-(t-T_{2k+3})}\frac{|x(T_{2k+3})|}{|y(T_{2k+3})|} \ge \delta (T_{2k+3}-T_{2k+2})e^{-(T_{2k+4}-T_{2k+3})} \ge \frac{\delta }{\varepsilon }, \end{aligned}$$

which shows (23).

To conclude the proof, we use the maximum norm in $\mathbb {R}^2$. With respect to this norm, $\Vert X(t)\xi \Vert =|x(t)|$, whenever $t\ge T_{2K+2}$. Our aim is to show that

25 $$\begin{aligned} e^{-(t-s)} \le \frac{\Vert X(t)\xi \Vert }{\Vert X(s)\xi \Vert } \le e^{(-1+\varepsilon )(t-s)} \quad \text {for all }\,t\ge s\ge T_{2K+2}\,. \end{aligned}$$

Equivalently, we prove (25) for $k\ge K+1$ and for $t,s\in [T_{2k},T_{2k+2}]$ such that $t\ge s$. For $T_{2k}\le s\le t\le T_{2k+1}$, we have

$$\begin{aligned} \frac{\Vert X(t)\xi \Vert }{\Vert X(s)\xi \Vert }&=\frac{|x(t)|}{|x(s)|} =e^{-(t-s)}\frac{x(T_{2k})+\delta (t-T_{2k})y(T_{2k})}{x(T_{2k})+\delta (s-T_{2k})y(T_{2k})}\\&=e^{-(t-s)}\frac{1+\delta (t-T_{2k})y(T_{2k})/x(T_{2k})}{1+\delta (s-T_{2k})y(T_{2k})/x(T_{2k})}\\&=e^{-(t-s)}\left( 1+\frac{\delta (t-s)y(T_{2k})/x(T_{2k})}{1+\delta (s-T_{2k})y(T_{2k})|/x(T_{2k})}\right) \,, \end{aligned}$$

which together with (23) implies that

$$\begin{aligned} e^{-(t-s)}\le \frac{\Vert X(t)\xi \Vert }{\Vert X(s)\xi \Vert } \le e^{-(t-s)}(1+\varepsilon (t-s)) \le e^{(-1+\varepsilon )(t-s)}\,. \end{aligned}$$

If $$T_{2k+1}\le s\le t\le T_{2k+2}$$, then

$$\begin{aligned} \frac{\Vert X(t)\xi \Vert }{\Vert X(s)\xi \Vert } = \frac{|x(t)|}{|x(s)|} = e^{-(t-s)}. \end{aligned}$$

This means that (25) is proved. Consequently, $\Sigma _{\xi }\subset [-1,-1+\varepsilon ]$. Letting $\varepsilon \rightarrow 0$ leads to $\Sigma _{\xi }=\{-1\}$ and finishes the proof of this proposition. $\square$

### Coincidence of the Bohl and Sacker–Sell Spectrum in Special Cases

We first show that the Bohl spectrum is a subset of the Sacker–Sell spectrum. We then show that the two spectra coincide when the Sacker–Sell spectral intervals are singletons. Finally, we show that the Bohl and Sacker–Sell spectra coincide for bounded diagonalizable, and hence, bounded integrally separated systems.

Let $\xi ,\eta \in \mathbb {R}^d\setminus \{0\}$. Then the two solutions $X(t)\xi$ and $X(t)\eta$ of (1) are said to be *integrally separated* if there exists $K\ge 1$ and $\alpha >0$ such that

26 $$\begin{aligned} \frac{\Vert X(t)\xi \Vert }{\Vert X(s)\xi \Vert } \ge Ke^{\alpha (t-s)} \frac{\Vert X(t)\eta \Vert }{\Vert X(s)\eta \Vert } \quad \text {for all }\,t\ge s\ge 0 \end{aligned}$$

(see e.g. [1, Definition 5.3.1]). If *A*(*t*) is bounded, then the angle between two such solutions is bounded below by a positive number.

In the next lemma, we show that when the solutions are integrally separated and $X(t)\xi$ is the bigger solution in the above sense, then the Bohl spectrum of any non-trivial linear combination of $X(t)\xi$ and $X(t)\eta$ is always given by $\Sigma _\xi$.

#### **Lemma 18**

Consider $\xi ,\eta \in \mathbb {R}^d\setminus \{0\}$ such that the two solutions $X(t)\xi$ and $X(t)\eta$ of (1) are integrally separated, i.e. the inequality (26) holds. Then

27 $$\begin{aligned} \Sigma _{\lambda \xi +\mu \eta }= \Sigma _{\xi }\quad \text {for all }\,\lambda \in \mathbb {R}\setminus \{0\}\text { and }\mu \in \mathbb {R}\,. \end{aligned}$$

#### *Proof*

The lemma is clear for $\mu =0$. For the rest, we may prove (27) for the case that $\lambda =\mu =1$. By taking $s=0$ in (26), there exists $T>0$ such that

$$\begin{aligned} \frac{\Vert X(t)\eta \Vert }{\Vert X(t)\xi \Vert }\le \frac{1}{2}\quad \text {for all }\,t\ge T\,. \end{aligned}$$

Thus, for all $t\ge T$, we have

28 $$\begin{aligned} \frac{1}{2}\le 1-\frac{\Vert X(t)\eta \Vert }{\Vert X(t)\xi \Vert } \le \frac{\Vert X(t)(\xi +\eta )\Vert }{\Vert X(t)\xi \Vert } \le 1+\frac{\Vert X(t)\eta \Vert }{\Vert X(t)\xi \Vert }\le \frac{3}{2}\,. \end{aligned}$$

By (28), for all $t\ge s\ge T$, we have

$$\begin{aligned} \frac{\Vert X(t)(\xi +\eta )\Vert }{\Vert X(s)(\xi +\eta )\Vert } = \frac{\Vert X(t)(\xi +\eta )\Vert }{\Vert X(t)\xi \Vert }\frac{\Vert X(t)\xi \Vert }{\Vert X(s)\xi \Vert }\frac{\Vert X(s)\xi \Vert }{\Vert X(s)(\xi +\eta )\Vert }\le 3\frac{\Vert X(t)\xi \Vert }{\Vert X(s)\xi \Vert }\,, \end{aligned}$$

which implies that

29 $$\begin{aligned} \frac{1}{t-s}\ln \frac{\Vert X(t)(\xi +\eta )\Vert }{\Vert X(s)(\xi +\eta )\Vert }\le \frac{\ln 3}{t-s}+\frac{1}{t-s}\ln \frac{\Vert X(t)\xi \Vert }{\Vert X(s)\xi \Vert }\,. \end{aligned}$$

Conversely, for all $t\ge s\ge T$, we have

$$\begin{aligned} \frac{\Vert X(t)(\xi +\eta )\Vert }{\Vert X(s)(\xi +\eta )\Vert } = \frac{\Vert X(t)(\xi +\eta )\Vert }{\Vert X(t)\xi \Vert }\frac{\Vert X(t)\xi \Vert }{\Vert X(s)\xi \Vert }\frac{\Vert X(s)\xi \Vert }{\Vert X(s)(\xi +\eta )\Vert }\ge \frac{1}{3}\frac{\Vert X(t)\xi \Vert }{\Vert X(s)\xi \Vert }\,, \end{aligned}$$

so that

30 $$\begin{aligned} \frac{1}{t-s}\ln \frac{\Vert X(t)(\xi +\eta )\Vert }{\Vert X(s)(\xi +\eta )\Vert }\ge -\frac{\ln 3}{t-s}+\frac{1}{t-s}\ln \frac{\Vert X(t)\xi \Vert }{\Vert X(s)\xi \Vert }\,. \end{aligned}$$

Let $\{t_n\}_{n\in \mathbb {N}}$ and $\{s_n\}_{n\in \mathbb {N}}$ be two positive sequences with $\lim _{n\rightarrow \infty } (t_n-s_n)=\infty$ and $\lim _{n\rightarrow \infty }s_n=\infty$. Since $\lim _{n\rightarrow \infty }t_n=\lim _{n\rightarrow \infty }s_n=\infty$, there exists $N\in \mathbb {N}$ such that $t_n\ge s_n\ge T$ for all $n\ge N$. Hence, combining (29) and (30) yields

$$\begin{aligned} \lim _{n\rightarrow \infty }\frac{1}{t_n-s_n}\ln \frac{\Vert X(t_n)(\xi +\eta )\Vert }{\Vert X(s_n)(\xi +\eta )\Vert } = \lim _{n\rightarrow \infty }\frac{1}{t_n-s_n}\ln \frac{\Vert X(t_n)\xi \Vert }{\Vert X(s_n)\xi \Vert }\,, \end{aligned}$$

whenever one of the two above limits exists. This fact, together with Lemma 9 (i), shows that $\Sigma _{\xi +\eta }=\Sigma _{\xi }$. This concludes the proof of this lemma. $\square$

We first use this lemma to show that the Bohl spectrum is a subset of the Sacker–Sell spectrum. As a consequence, the filtration corresponding to the Bohl spectrum is finer than the filtration corresponding to Sacker–Sell spectrum.

#### **Theorem 19**

Consider the Bohl spectrum $\Sigma _\mathrm{Bohl}$ and the Sacker–Sell spectrum $\Sigma _\mathrm{SS}$ of a linear nonautonomous differential equation (1). The following statements hold:

(i) The Bohl spectrum is a subset of the Sacker–Sell spectrum.
(ii) The filtration associated with the Bohl spectrum is finer than the one of Sacker–Sell spectrum.

#### *Proof*

(i) Let $\lambda \in \mathbb {R}\setminus \Sigma _\mathrm{SS}$ be arbitrary. Then $\dot{x} = A(t)x$ has an exponential dichotomy with growth rate $\lambda$, which means that there exists a projector $P\in \mathbb {R}^{d\times d}$ such that

31 $$\begin{aligned} \Vert X(t)PX^{-1}(s)\Vert \le Ke^{(\lambda -\alpha )(t-s)}\quad \text {for all }\,0\le s\le t \end{aligned}$$

and

32 $$\begin{aligned} \Vert X(t)(\mathbbm {1}-P)X^{-1}(s)\Vert \le Ke^{-(\lambda +\alpha )(s-t)}\quad \text {for all }\,0\le t\le s\,. \end{aligned}$$

Then for any $\xi \in \ker P\setminus \{0\}$ and $\eta \in \hbox { im}\, P\setminus \{0\}$, the solutions $X(t)\xi$ and $X(t)\eta$ are integrally separated. So, by virtue of Lemma 18, we have

$$\begin{aligned} \Sigma _\mathrm{Bohl} = \bigcup _{\xi \in \ker P\setminus \{0\}}\Sigma _{\xi } \cup \bigcup _{\eta \in {\text {im}}\, P\setminus \{0\}}\Sigma _{\eta }\,. \end{aligned}$$

From (31), we derive that for all $\eta \in \hbox { im}\, P\setminus \{0\}$,

$$\begin{aligned} \Vert X(t)\eta \Vert =\Vert X(t)P\eta \Vert \le Ke^{(\lambda -\alpha )(t-s)}\Vert X(s)\eta \Vert \,, \end{aligned}$$

which implies that $\Sigma _{\eta }\subset (-\infty ,\lambda -\alpha ]$. Similarly, using (32), we obtain that if $\xi$ is in the kernel of *P*, then $\Sigma _{\xi }\subset [\lambda +\alpha ,\infty )$. Thus, $\lambda \not \in \Sigma _\mathrm{Bohl}$, which finishes the proof of (i).

(ii) Let $I_j$ be the rightmost component of the Bohl spectrum contained in Sacker–Sell spectral interval $[a_i,b_i]$, and let ${\mathcal {S}}_j$ be the subspace in the Bohl filtration corresponding to the union of $I_j$ and the intervals to its left. We only treat the case that $[a_i,b_i]$ is not the last Sacker–Sell spectral interval and leave the other case to the reader. Then ${\mathcal {S}}_j=\mathcal {M}_{\lambda }=\big \{\xi \in \mathbb {R}^d: \Sigma _{\xi }\subset [-\infty ,\lambda )\big \}$ for $\lambda \in (b_i,a_{i+1})$, where $\mathcal {M}_\lambda$ is defined as in (11). If $\xi \in S_j$, then for each $\lambda \in (b_i,a_{i+1})$, there exists $K\ge 1$ such that

$$\begin{aligned} \Vert X(t)\xi \Vert \le Ke^{\lambda (t-s)}\Vert X(s)\xi \Vert \quad \text {for all }\,t\ge s\,. \end{aligned}$$

It follows that $\xi$ is in the pseudo-stable subspace for the exponential dichotomy with growth rate $\lambda$ of $\dot{x}=A(t)x$ for $\lambda \in (b_i,a_{i+1})$. Hence, $\xi \in {\mathcal {W}}_i$, which proves ${\mathcal {S}}_{j}\subset {\mathcal {W}}_{i}$, with ${\mathcal {W}}_i$ defined as in Theorem 4. Conversely, note that since ${\mathcal {W}}_{i}$ is the pseudo-stable subspace for the exponential dichotomy with growth rate $\lambda$ of $\dot{x}=A(t)x$ for $\lambda \in (b_i,a_{i+1})$, there exist constants $K, \alpha >0$ such that for all $\xi \in {\mathcal {W}}_{i}$, we have

$$\begin{aligned} \Vert X(t)\xi \Vert \le Ke^{(\lambda -\alpha )(t-s)}\Vert X(s)\xi \Vert \quad \text {for all }\,t\ge s\,. \end{aligned}$$

This means that $\Sigma _{\xi }\subset (-\infty ,\lambda )$ for all $\lambda \in (b_i,a_{i+1})$ which implies that $\xi \in {\mathcal {S}}_{j}$. Thus ${\mathcal {S}}_{j}={\mathcal {W}}_{i}$. In fact, what we have proved is that the Bohl filtration is ${\mathcal {S}}_{i}$, $i\in \{1,\dots ,m\}$, and the Sacker–Sell filtration is ${\mathcal {W}}_{i}$, for $i\in \{1,\ldots ,n\}$, where $n\le m\le d$, and there exist $i_{1}<i_{2}<\cdots <i_{n}$ such that ${\mathcal {S}}_{i_{j}}={\mathcal {W}}_{j}$.

#### **Theorem 20**

(Bohl and Sacker–Sell spectra of diagonalizable systems) Suppose that the bounded linear nonautonomous differential equation (1) is *diagonalizable*, i.e. it is kinematically similar to a (nonautonomous) diagonal system. Then the Bohl and Sacker–Sell spectrum of (1) coincide. In particular, both spectra coincide for bounded one-dimensional systems.

#### *Proof*

By assumption, the linear system (1) is kinematically similar to a diagonal system

33 $$\begin{aligned} \dot{x}=D(t)x=\mathrm{diag}(a_1(t),\ldots , a_d(t))\,x\,, \end{aligned}$$

where the $a_i(t)$ are bounded. Since the Bohl and Sacker–Sell spectra are invariant under kinematic similarity, it is sufficient to show that the Bohl spectrum $\Sigma _\mathrm{Bohl}$ and the dichotomy spectrum $\Sigma _\mathrm{SS}$ of (33) coincide. For $i\in \{1,\dots , d\}$, define

$$\begin{aligned} \alpha _i:=\liminf _{t-s\rightarrow \infty }\frac{1}{t-s}\int ^t_sa_{i}(u)\,\mathrm {d}u \quad \text {and}\quad \beta _i=\limsup _{t-s\rightarrow \infty }\frac{1}{t-s}\int ^t_sa_{i}(u)\,\mathrm {d}u\,. \end{aligned}$$

It follows from Proposition 5 that

$$\begin{aligned} \Sigma _\mathrm{SS}=\bigcup _{i=1}^d\,[\alpha _i,\beta _i]\,. \end{aligned}$$

To compute $\Sigma _\mathrm{Bohl}$, let $(e_1,\dots ,e_d)$ denote the standard orthonormal basis of $\mathbb {R}^d$. A simple computation yields that

$$\begin{aligned} \Sigma _{e_i}=[\alpha _i,\beta _i]\quad \text {for all }\,i\in \{1,\dots ,d\}\,, \end{aligned}$$

which implies

$$\begin{aligned} \bigcup _{i=1}^d[\alpha _i,\beta _i]\subset \Sigma _\mathrm{Bohl}\subset \Sigma _\mathrm{SS} =\bigcup _{i=1}^d[\alpha _i,\beta _i]\,, \end{aligned}$$

and completes the proof. $\square$

#### *Remark 21*

Proposition 16 and Theorem 20 also show that the Bohl spectrum of a bounded upper triangular system is, in general, not equal to that for the diagonal part, unlike the situation for the Sacker–Sell spectrum in the bounded half-line case (see also Proposition 5). However the Bohl spectrum of the triangular system is a subset of the Sacker–Sell spectrum (see Theorem 19 above), which equals the Sacker–Sell spectrum of the diagonal part, and the Sacker–Sell spectrum of the diagonal part coincides with its Bohl spectrum (see Theorem 20 above). We conclude that for bounded systems, the Bohl spectrum of an upper triangular system is a subset of the Bohl spectrum of its diagonal part.

The linear nonautonomous differential equation (1) is said to be *integrally separated* if there are *d* independent solutions $X(t)\xi _1,\dots ,X(t)\xi _d$ such that $X(t)\xi _i$ and $X(t)\xi _{i+1}$ are integrally separated for all $i\in \{1,\dots ,d-1\}$.

We now prove using the previous theorem that the Bohl and Sacker–Sell spectra coincide for bounded integrally separated systems. This means also that the Bohl spectrum depends continuously on parameters for such systems.

#### **Corollary 22**

Suppose that system (1) is integrally separated, and *A*(*t*) is bounded in $t\in \mathbb {R}^+_0$. Then the Bohl spectrum coincides with the Sacker–Sell spectrum of (1).

#### *Proof*

By Bylov’s Theorem [1, Theorem 5.3.1, p. 149], the linear system (1) is kinematically similar to a bounded diagonal system

$$\begin{aligned} \dot{x}=D(t)x=\mathrm{diag}(a_1(t),\ldots , a_d(t))x\,, \end{aligned}$$

and a direct application of Theorem 20 completes the proof. $\square$

#### *Remark 23*

The boundedness assumption of *A*(*t*) in the above corollary is needed, since there exists an unbounded integrally separated system which is not diagonalizable such that its Bohl spectrum and and its Sacker–Sell spectrum are different. Consider the system $\dot{x}=A(t)x$, where *A*(*t*) is defined by

$$\begin{aligned} A(t) := \begin{pmatrix} 0 &{} 2e^{t} \\ 0 &{} 1 \\ \end{pmatrix} \quad \text {for all }\,t \ge 0\,. \end{aligned}$$

The fundamental matrix solution *X*(*t*) of this system is given by

$$\begin{aligned} X(t)= \begin{pmatrix} 1 &{} e^{2t}-1 \\ 0 &{} e^t \\ \end{pmatrix} \quad \text {for all }\,t \ge 0\,. \end{aligned}$$

Note that

$$\begin{aligned} X(t)\begin{pmatrix} 1 \\ 0 \end{pmatrix} = \begin{pmatrix} 1\\ 0\\ \end{pmatrix} \quad \text {and}\quad X(t) \begin{pmatrix} 0 \\ 1 \\ \end{pmatrix} = \begin{pmatrix} e^{2t}-1\\ e^t\\ \end{pmatrix} , \end{aligned}$$

which implies that these two solutions are integrally separated. It follows from Lemma 18 that $\Sigma _{\mathrm{Bohl}}=\{0\}\cup \{2\}$, and by explicit presentation of *X*(*t*), we see that the system is not reducible and hence $\Sigma _{\mathrm{SS}}$ is an interval containing the points 0 and 2.

Let ${\mathcal {B}}$ denote the linear space of bounded measurable matrix-valued functions $A:\mathbb {R}_0^+\rightarrow \mathbb {R}^{d\times d}$. We endow ${\mathcal {B}}$ with the $L^{\infty }$-norm defined by

$$\begin{aligned} \Vert A-B\Vert _{\infty }={\text {ess sup}}_{t\in \mathbb {R}_0^+}\Vert A(t)-B(t)\Vert \,, \end{aligned}$$

so that $({\mathcal {B}},\Vert \cdot \Vert _{\infty })$ is a Banach space. Using [24], one can show that there exists an open and dense set ${\mathcal {R}}$ of ${\mathcal {B}}$ such that for all $A\in {\mathcal {R}}$, the associated linear nonautonomous differential equation is integrally separated (note that genericity of exponential dichotomies for two-dimensional quasi-periodic linear systems was treated in [15]). As a consequence, we obtain the following corollary.

#### **Corollary 24**

(Coincidence is generic) The Bohl spectrum and the Sacker–Sell spectrum coincide generically for bounded linear nonautonomous differential equations.

We demonstrate by means of a counterexample that the Bohl spectrum in not even upper semi-continuous in general with perturbations to the right-hand side in the $L^{\infty }$-norm. Note that the Sacker–Sell spectrum is upper semi-continuous in general, and in [26], sufficient criteria for continuity of the Sacker–Sell spectrum are established.

#### **Corollary 25**

(Discontinuity of the Bohl spectrum) The mapping $A\mapsto \Sigma _\mathrm{Bohl}(A)$ is not upper semi-continuous in general.

#### *Proof*

Consider the linear system (20), and for $\varepsilon \in \mathbb {R}$, define the perturbations

$$\begin{aligned} A_{\varepsilon }(t):= \begin{pmatrix} -1&{}\delta \\ 0&{}-1+\varepsilon \end{pmatrix} \quad \text {for all }\,t\in [T_{2k}, T_{2k+1}] \end{aligned}$$

and

$$\begin{aligned} A_{\varepsilon }(t) := \begin{pmatrix}-1&{}0\\ 0&{}\varepsilon \end{pmatrix} \quad \text {for all }\,t\in [T_{2k+1}, T_{2k+2}]\,. \end{aligned}$$

Looking at the diagonal, we see that this system has the Sacker–Sell spectrum $\{-1\}\cup [-1+\varepsilon ,\varepsilon ]$. In particular, for $\varepsilon >0$, it follows that the system is integrally separated, and hence, the Bohl spectrum is also $\{-1\}\cup [-1+\varepsilon ,\varepsilon ]$. However, the Bohl spectrum for $\varepsilon = 0$ is given by $\{-1\}$ (see Proposition 16), so the Bohl spectrum is not upper semi-continuous at $\varepsilon = 0$. $\square$

Suppose the Sacker–Sell spectrum consists of points. Then by Theorem 19, the Bohl spectrum consists of points. We still need to prove each point in the Sacker–Sell spectrum is also in the Bohl spectrum. This follows from the next lemma.

#### **Lemma 26**

Let [*a*, *b*] be a spectral interval of the Sacker–Sell spectrum of the linear nonautonomous differential equation (1). Then there exists a solution whose Bohl spectrum is contained in [*a*, *b*].

#### *Proof*

Let $\{I_i=[a_i,b_i]\}_{i\in \{1,\ldots ,k\}}$ be the ordered Sacker–Sell spectral intervals of (1). Consider the filtration

$$\begin{aligned} \{0\}={\mathcal {W}}_0 \subsetneq {\mathcal {W}}_1\subsetneq {\mathcal {W}}_2\subsetneq \dots \subsetneq {\mathcal {W}}_k =\mathbb {R}^d\,, \end{aligned}$$

established in Theorem 4, satisfying the dynamical characterization

$$\begin{aligned} {\mathcal {W}}_i= \Big \{\textstyle \xi \in \mathbb {R}^d: \sup _{t\in \mathbb {R}^+_0} \Vert X(t)\xi \Vert e^{-\gamma t} < \infty \Big \} \end{aligned}$$

for all $i\in \{1,\dots ,k-1\}$ and $\gamma \in (b_i,a_{i+1})$. There exists an $i\in \{1,\dots ,k\}$ such that $I_i = [a,b] = [a_i,b_i]$. Note that ${\mathcal {W}}_{i-1}$ is a proper subspace of ${\mathcal {W}}_i$ and we can write ${\mathcal {W}}_i={\mathcal {W}}_{i-1}\oplus \mathcal V$ with $\mathcal V \not = \{0\}$. Now $\dot{x}=A(t)x$ has an exponential dichotomy with growth rate $b+\varepsilon$ with pseudo-stable subspace ${\mathcal {W}}_i$. This means that for all $\xi \in {\mathcal {W}}_i$, there exist $K_1>0$ and $\alpha _1>0$ such that

34 $$\begin{aligned} \frac{\Vert X(t)\xi \Vert }{\Vert X(s)\xi \Vert } \le K_1e^{(b+\varepsilon -\alpha _1)(t-s)} \quad \text {for all }\,t\ge s\ge 0\,. \end{aligned}$$

Next $\dot{x}=A(t)x$ has an exponential dichotomy with growth rate $a-\varepsilon$ with a pseudo-unstable subspace $\mathcal V$ [27, Remark 5.6 and Lemma 6.1]. This means that for all $\xi \in {\mathcal {V}}$, there exist $K_2>0$ and $\alpha _2>0$ such that

35 $$\begin{aligned} \frac{\Vert X(t)\xi \Vert }{\Vert X(s)\xi \Vert } \ge K_2e^{(a-\varepsilon +\alpha _2)(t-s)} \quad \text {for all }\,t\ge s\ge 0\,. \end{aligned}$$

From (34), it follows that

$$\begin{aligned} \limsup _{t-s\rightarrow \infty }\frac{1}{t-s}\ln \frac{\Vert X(t)\xi \Vert }{\Vert X(s)\xi \Vert } \le b+\varepsilon -\alpha _1<b+\varepsilon \,, \end{aligned}$$

and from (35), it follows that

$$\begin{aligned} \liminf _{t-s\rightarrow \infty }\frac{1}{t-s}\ln \frac{\Vert X(t)\xi \Vert }{\Vert X(s)\xi \Vert } \ge a-\varepsilon +\alpha _2>a-\varepsilon \,. \end{aligned}$$

Since $\varepsilon >0$ was chosen arbitrarily, it follows that

$$\begin{aligned} a\le \liminf _{t-s\rightarrow \infty }\frac{1}{t-s}\ln \frac{\Vert X(t)\xi \Vert }{\Vert X(s)\xi \Vert } \le \limsup _{t-s\rightarrow \infty }\frac{1}{t-s}\ln \frac{\Vert X(t)\xi \Vert }{\Vert X(s)\xi \Vert } \le b\,. \end{aligned}$$

Thus, $$\Sigma _{\xi }\subset [a,b]$$. $$\square $$

#### **Corollary 27**

If the Sacker–Sell spectrum consists of points, then it coincides with the Bohl spectrum.

#### *Remark 28*

Each component of the Sacker–Sell spectrum contains points of the Bohl spectrum. One may ask how many components of the Bohl spectrum can there be in a Sacker–Sell spectral interval. For a bounded integrally separated system, the answer is one since the two spectra coincide. For bounded systems in two dimensions, that leaves us with the case where the Sacker–Sell spectrum is one interval, and the system is not integrally separated. Then if the Bohl spectrum had two components, we would have two integrally separated solutions. So there can only be one component. However in three dimensions, consider the system

$$\begin{aligned} \dot{x}=a(t)x,\quad \dot{y}=A(t)y, \end{aligned}$$

where the first is a scalar system with Bohl spectrum equal to the Sacker–Sell spectrum, given by $\big [-\frac{1}{2},\frac{1}{2}\big ]$ and the second is the two-dimensional system, we constructed in Sect. 5.1 with Sacker–Sell spectrum $=[-1,0]$ and Bohl spectrum $\{-1\}$. Then the three-dimensional system has Sacker–Sell spectrum $\big [-1,\frac{1}{2}\big ]$, but the Bohl spectrum is given by $\big [-\frac{1}{2},\frac{1}{2}\big ] \cup \{-1\}$, where we have used Lemma 18.

## Nonlinear Perturbations

This section is devoted to study whether the trivial solution of a nonlinearly perturbed system with negative Bohl spectrum is asymptotically stable. Note that if the Sacker–Sell spectrum is negative, then nonlinear stability follows directly, but we will show below by means of a counter example that we cannot obtain such a result for the Bohl spectrum. Before doing so, we look at the example from Sect. 5.1 with negative Bohl spectrum, and we prove that the system is exponentially stable for any nonlinear perturbation. Since the Sacker–Sell spectrum of this linear system is not negative, this shows that even in those cases, stability for the nonlinear system can follow. Despite the fact that negative Bohl spectrum does not imply nonlinear stability, in a forthcoming paper, we will discuss additional conditions on the nonlinearity that guarantee nonlinear stability for systems with negative Bohl spectrum, which include cases where the Sacker–Sell spectrum cannot indicate stability.

### **Proposition 29**

Consider the nonlinear differential equation

36 $$\begin{aligned} \dot{x} = A(t)x+ f(t,x)\,, \end{aligned}$$

where $A:\mathbb {R}^+_0\rightarrow \mathbb {R}^{d\times d}$ is given as in (19), and $f:\mathbb {R}^+_0\times \mathbb {R}^d\rightarrow \mathbb {R}^d$ is continuous with

$$\begin{aligned} \Vert f(t,x)\Vert \le L\Vert x\Vert ^q \quad \text {for all }\,t\in \mathbb {R}^+_0\text { and }x\in B_\delta (0) \end{aligned}$$

for some $\delta >0$, $L\ge 1$ and $q>1$. Then the trivial solution of (36) is exponentially stable, i.e. there exist $\alpha > 0$ and ${\tilde{\delta }} > 0$ such that

$$\begin{aligned} \Vert \varphi (t,0,x)\Vert \le K e^{-\alpha t}\Vert x\Vert \quad \text {for all }\,t\in \mathbb {R}^+_0 \text { and }x\in B_{{\tilde{\delta }}}(0)\,, \end{aligned}$$

where $\varphi$ denotes the general solution of (36).

### *Proof*

Since the Bohl spectrum is given by $\{-1\}$, both Lyapunov exponents must be $-1$. However, the sum of the Lyapunov exponents is bounded below by

$$\begin{aligned} \limsup _{t\rightarrow \infty }\frac{1}{t}\int ^t_0\mathrm{Tr}\,A(u)\,\mathrm {d}u\,, \end{aligned}$$

see [1, Theorem 2.5.1] and [8, p. 226]. For our system, this means

$$\begin{aligned} -2 \ge \limsup _{t\rightarrow \infty }\frac{1}{t}\int ^t_0 (-1+a_{22}(u))\,\mathrm {d}u\,, \end{aligned}$$

and hence that

$$\begin{aligned} -1\ge \limsup _{t\rightarrow \infty }\frac{1}{t}\int ^t_0 a_{22}(u)\,\mathrm {d}u\,. \end{aligned}$$

On the other hand, since $a_{22}(t)\ge -1$ for all $t\ge 0$, it follows that

$$\begin{aligned} -1 \le \liminf _{t\rightarrow \infty }\frac{1}{t}\int ^t_0 a_{22}(u)\,\mathrm {d}u\,. \end{aligned}$$

We conclude that

$$\begin{aligned} \lim _{t\rightarrow \infty }\frac{1}{t}\int ^t_0 a_{22}(u)\,\mathrm {d}u=-1 \end{aligned}$$

and so we get regularity from [16, Theorem 64.2] or [1, Theorem 3.8.1]. Then our system is regular and has negative Lyapunov exponents, so for any higher-order perturbation, the zero solution is exponentially stable (see [21], [16, Theorem 65.3] or [7]).

### *Remark 30*

Consider the linear system (20) used in the above proposition, and let $a<b$. Then the system

$$\begin{aligned} \dot{x}=\big ((b-a)A((b-a)t)+b\big )x \end{aligned}$$

has Sacker–Sell spectrum [*a*, *b*] and Bohl spectrum $\{a\}$ since either spectrum of $\dot{x}=\gamma A(\gamma t)x$ is $\gamma$ times the spectrum of $\dot{x}=A(t)x$, and the spectrum of $\dot{x}=(A(t)+b)x$ is the translation of the spectrum of $\dot{x}=A(t)x$ by the number *b*. Taking $[a,b] = [-1+\varepsilon ,\varepsilon ]$, where $0<\varepsilon <1$, implies that we get a system with $\Sigma _\mathrm{Bohl}=\{-1+\varepsilon \}$ and $\Sigma _\mathrm{SS} = [-1+\varepsilon ,\varepsilon ]$, and we obtain asymptotic stability for nonlinear perturbations similarly to Proposition 29, although the Sacker–Sell spectrum has nontrivial intersection with the position half line.

We now study an example for which the Bohl spectrum is negative, and there exists a nonlinear perturbation such that the trivial solution of the nonlinear system is not asymptotically stable. Let $\alpha ,\beta ,\gamma$ and $\delta$ be positive real numbers with

37 $$\begin{aligned} \beta >2\gamma +3\alpha \,,\, \gamma >2\alpha \quad \text {and}\quad \delta \ge 1\,. \end{aligned}$$

Define a piecewise constant matrix-valued function $A:\mathbb {R}_0^+\rightarrow \mathbb {R}^{2\times 2}$ by

38 $$\begin{aligned} A(t):=\left\{ \begin{array}{ccl} A_1 &{} : &{} t\in [0,1) \hbox { or } 2^{2k+1}\le t < 2^{2k+2}\,, \\ A_2 &{} : &{} 2^{2k}\le t < 2^{2k+1}\,, \end{array}\right. \end{aligned}$$

where $$k\in \mathbb {N}_0$$ and

$$\begin{aligned} A_1:=\left( \begin{matrix} -\alpha &{} \delta \\ 0&{}-\beta \end{matrix}\right) \quad \text {and}\quad A_2:=\left( \begin{matrix} -\alpha &{} \delta \\ 0&{}\gamma \end{matrix}\right) \,. \end{aligned}$$

We now compute the Bohl spectrum of the system

39 $$\begin{aligned} \dot{x}=A(t)x\,, \end{aligned}$$

where $A:\mathbb {R}_0^+\rightarrow \mathbb {R}^{2\times 2}$ is defined as in (38). We need the following preparatory result.

### **Lemma 31**

Let (*x*(*t*), *y*(*t*)) be a solution of (39). Then

$$\begin{aligned} |y(t)| \le e^{-\frac{\beta -2\gamma }{3}t}|y(0)| \quad \text {for all }\,t\ge 0\,. \end{aligned}$$

### *Proof*

For any $k\in \mathbb {N}_0$, we have

$$\begin{aligned} y(2^{2k+2})= e^{-\beta 2^{2k+1}} y(2^{2k+1}) \quad \text {and}\quad y(2^{2k+1})= e^{\gamma 2^{2k} } y(2^{2k})\,, \end{aligned}$$

which implies that

$$\begin{aligned} y(2^{2k+2}) = e^{-(2\beta -\gamma )2^{2k}}y(2^{2k}). \end{aligned}$$

Hence,

$$\begin{aligned} y(2^{2k})&= e^{-(2\beta -\gamma )(2^{2k-2}+\dots + 2^0)}y(1)\\&= e^{-\frac{2\beta -\gamma }{3}2^{2k}}e^{-\frac{\beta +\gamma }{3}} y(0)\,, \end{aligned}$$

and

$$\begin{aligned} y(2^{2k+1}) = e^{2^{2k}\gamma }y(2^{2k}) = e^{-\frac{\beta -2\gamma }{3}2^{2k+1}}e^{-\frac{\beta +\gamma }{3}} y(0). \end{aligned}$$

Consequently, if $2^{2k}\le t < 2^{2k+1}$, then

$$\begin{aligned} |y(t)|&= e^{\gamma (t-2^{2k})} |y(2^{2k})|\\&= e^{\gamma (t-2^{2k})} e^{-\frac{2\beta -\gamma }{3}2^{2k}}e^{-\frac{\beta +\gamma }{3}} |y(0)|\\&\le e^{-\frac{\beta -2\gamma }{3}t}|y(0)|, \end{aligned}$$

and if $2^{2k+1}\le t < 2^{2k+2}$, then

$$\begin{aligned} |y(t)|&= e^{-\beta (t-2^{2k+1})} |y(2^{2k+1})|\\&= e^{-\beta (t-2^{2k+1})} e^{-\frac{\beta -2\gamma }{3}2^{2k+1}}e^{-\frac{\beta +\gamma }{3}} |y(0)|.\\&\le e^{-\frac{\beta -2\gamma }{3}t}|y(0)|, \end{aligned}$$

which completes the proof of this lemma. $\square$

### **Proposition 32**

The Bohl spectrum of (39) satisfies $\Sigma _\mathrm{Bohl}\le -\alpha <0$.

### *Proof*

Fix an initial condition $(x_0,y_0)\in \mathbb {R}^2$, and let $\xi (t)=(x(t),y(t))^{\mathrm {T}}$ denote the solution of (39) with $\xi (0)=(x_0,y_0)^{\mathrm {T}}$. Obviously, $\Sigma _{\xi }=\Sigma _{-\xi }$ and we thus may assume that $y_0\ge 0$. Let $\mathbb {R}^2$ be endowed with the maximum norm for the remainder of this proof. We consider the following two cases.

*Case* 1 $y_0=0$. Then we have $\xi (t)=(e^{-\alpha t},0)^{\mathrm {T}}$, which implies $\Sigma _{\xi }=\{-\alpha \}$.

*Case* 2 $y_0\not =0$. By the variation of constants formula, we have

$$\begin{aligned} x(t)=e^{-\alpha t}\left( x_0+\delta \int _0^t e^{\alpha s} y(s)\,\mathrm {d}s\right) \,, \end{aligned}$$

and by (37) and Lemma 31, the integral $\int _0^{\infty }e^{\alpha s} y(s)\,\mathrm {d}s$ exists. We divide the remainder of Case 2 into two different cases.

*Case* 2.1 $$x_0\not =-\delta \int _0^{\infty }e^{\alpha s} y(s)\,\mathrm {d}s$$. Then

$$\begin{aligned} \lim _{t\rightarrow \infty } e^{\alpha t} |x(t)|= \left| x_0+\delta \int _0^{\infty } e^{\alpha s} y(s)\,\mathrm {d}s\right| \,. \end{aligned}$$

Hence, there exists a $T>0$ such that for all $t\ge T$, we have $|x(t)|>|y(t)|$. This implies

$$\begin{aligned} \lim _{t-s\rightarrow \infty }\frac{1}{t-s}\ln \frac{\Vert \xi (t)\Vert }{\Vert \xi (s)\Vert }=-\alpha \,, \end{aligned}$$

which leads to $\Sigma _{\xi }=\{-\alpha \}$.

*Case* 2.2 $$x_0=-\delta \int _0^{\infty }e^{\alpha s} y(s)\,\mathrm {d}s$$. Then

40 $$\begin{aligned} x(t)=-\delta e^{-\alpha t}\int _{t}^{\infty }e^{\alpha s} y(s)\,\mathrm {d}s<0\,. \end{aligned}$$

Since $y(t)>0$ for all $t\ge 0$, it follows that for $t\ge s$

41 $$\begin{aligned} e^{\alpha t} |x(t)| =\delta \int _t^{\infty } e^{\alpha u} y(u)\,\mathrm {d}u\le \delta \int _s^{\infty } e^{\alpha u} y(u)\,\mathrm {d}u= e^{\alpha s} |x(s)|\,. \end{aligned}$$

Having done this for the *x*-component of $\xi$, we now compare $e^{\alpha t}\Vert \xi (t)\Vert$ and $e^{\alpha s}\Vert \xi (s)\Vert$ with $t\ge s$. The following statements hold.

(i) For all $t,s\in [2^{2k+1}, 2^{2k+2})$ with $t\ge s$, we have $e^{\alpha t}|y(t)|\le e^{\alpha s} |y(s)|$, and with (41), we get
  $$\begin{aligned} \Vert \xi (t)\Vert \le e^{-\alpha (t-s)}\Vert \xi (s)\Vert \,. \end{aligned}$$
(ii) For all $t,s\in [2^{2k}, 2^{2k+1}-1)$ with $t\ge s$ and $k\in \mathbb {N}$, note that in the interval $[2^{2k}, 2^{2k+1}]$ the function *y*(*t*) is increasing, so we have
  $$\begin{aligned} e^{\alpha t}|x(t)|=\delta \int _t^{\infty } e^{\alpha s} y(s)\,\mathrm {d}s \ge \int _t^{2^{2k+1}} e^{\alpha s} y(s)\,\mathrm {d}s \ge e^{\alpha t} y(t) \end{aligned}$$
  for all $t,s\in [2^{2k}, 2^{2k+1}-1)$ with $t\ge s$. Hence, $\Vert \xi (t)\Vert =|x(t)|$, and from (41), we obtain
  $$\begin{aligned} \Vert \xi (t)\Vert \le e^{-\alpha (t-s)}\Vert \xi (s)\Vert \,. \end{aligned}$$
(iii) For $2^{2k+1}-1\le s \le t\le 2^{2k+1}$, we have
  $$\begin{aligned} \Vert \xi (t)\Vert \le e^{M(t-s)}\Vert \xi (s)\Vert \,, \end{aligned}$$
  where $M=\max \{\alpha +\delta ,\gamma \}$ is the operator norm of $A_2$ with respect to the maximum norm. In particular,
  42 $$\begin{aligned} \frac{\Vert \xi (2^{2k+1})\Vert }{\Vert \xi (2^{2k})\Vert } = \frac{\Vert \xi (2^{2k+1})\Vert }{\Vert \xi (2^{2k+1}-1)\Vert } \frac{\Vert \xi (2^{2k+1}-1)\Vert }{\Vert \xi (2^{2k})\Vert } \le e^{M+\alpha } e^{-\alpha (2^{2k+1}-2^{2k})}\,. \end{aligned}$$

Let $t> s\ge 4$. We choose $m,n\in \mathbb {N}$ such that

$$\begin{aligned} 2^{m+1}>t\ge 2^m\ge 2^n\ge s > 2^{n-1}\,. \end{aligned}$$

Note that

$$\begin{aligned} \frac{\Vert \xi (t)\Vert }{\Vert \xi (s)\Vert } = \frac{\Vert \xi (t)\Vert }{\Vert \xi (2^m)\Vert } \frac{\Vert \xi (2^n)\Vert }{\Vert \xi (s)\Vert } \prod _{k=n}^{m-1} \frac{\Vert \xi (2^{k+1})\Vert }{\Vert \xi (2^k)\Vert }\,. \end{aligned}$$

Using (42), we obtain

$$\begin{aligned} \prod _{k=n}^{m-1} \frac{\Vert \xi (2^{k+1})\Vert }{\Vert \xi (2^k)\Vert }&\le \prod _{k=n}^{m-1} e^{M+\alpha } e^{-\alpha (2^{2k+1}-2^{2k})}\\&= e^{(m-n)(M+\alpha )} e^{-\alpha (2^m-2^n)}\,. \end{aligned}$$

Analogously to (42), we have

$$\begin{aligned} \frac{\Vert \xi (t)\Vert }{\Vert \xi (2^m)\Vert } \le e^{M+\alpha } e^{-\alpha (t-2^m)} \quad \text {and}\quad \frac{\Vert \xi (2^n)\Vert }{\Vert \xi (s)\Vert } \le e^{M+\alpha } e^{-\alpha (2^n-s)}\,. \end{aligned}$$

Consequently, we obtain the estimate

$$\begin{aligned} \frac{\Vert \xi (t)\Vert }{\Vert \xi (s)\Vert } \le e^{(m-n+2)(M+\alpha )}e^{-\alpha (t-s)}\,. \end{aligned}$$

Thus,

$$\begin{aligned} \limsup _{t-s\rightarrow \infty }\frac{1}{t-s}\ln \frac{\Vert \xi (t)\Vert }{\Vert \xi (s)\Vert }&\le -\alpha +(M+\alpha )\limsup _{t-s\rightarrow \infty }\frac{\log _2(t/s)}{t-s}\\&\le -\alpha +(M+\alpha )\limsup _{t-s\rightarrow \infty }\frac{\log _2(1+(t-s)/4)}{t-s}= -\alpha \,, \end{aligned}$$

which completes the proof. $\square$

The following proposition shows that, although the Bohl spectrum is bounded above by $-\alpha <0$, for certain nonlinear perturbation of (39), the system is unstable.

### **Proposition 33**

Consider the perturbed system

43 $$\begin{aligned} \begin{pmatrix} \dot{x}\\ \dot{y} \end{pmatrix} = A(t) \begin{pmatrix} x\\ y \end{pmatrix} + \begin{pmatrix} 0\\ x^2 \end{pmatrix} \,, \end{aligned}$$

where *A*(*t*) is defined as in (38). Then the trivial solution of (43) is unstable.

### *Proof*

Let $(x_0,y_0)$ be an initial condition at time $t=0$ for the solution (*x*(*t*), *y*(*t*)) with $x_0>0$ and $y_0>0$. We prove $\limsup _{t\rightarrow \infty }y(t)=\infty$ with the following two steps.

*Step* 1 We show that both *x*(*t*) and *y*(*t*) are positive for all $t\ge 0$. If we suppose the contrary, then we can define

$$\begin{aligned} T^*:= \inf \big \{ t \ge 0: x(t)<0 \hbox { or } y(t)< 0 \big \} \end{aligned}$$

By continuity, we derive that $T^*>0$ and $x(t), y(t)>0$ for all $t\in [0,T^*)$. We now consider two cases.

*Case* 1 If $x(T^*)=0$, then by variation of constants formula we have

$$\begin{aligned} x(T^*)= e^{-\alpha T^*}x_0+ \delta \int _{0}^{T^*} e^{-\alpha (T^*-s)} y(s)\,\mathrm {d}s>0\,, \end{aligned}$$

which leads to a contradiction.

*Case* 2 If $y(T^*)=0$, then $\dot{y}(T^*)= x(T^*)^2>0$. Thus, there exists $\varepsilon >0$ such that $y(T^*-\varepsilon )<0$. This contradicts the definition of $T^*$.

*Step* 2 We estimate *y*(*t*). From the variation of constants formula for the first component, we have

$$\begin{aligned} x(t)\ge e^{-\alpha t} x_0\qquad \hbox {for all } t \ge 0\,. \end{aligned}$$

By variation of constants formula of the second component, we have

$$\begin{aligned} y(t)=\Lambda _2(t,0) y_0+\int _{0}^t \Lambda _2(t,s)x(s)^2\,\mathrm {d}s\,, \end{aligned}$$

where $\Lambda _2(t,s)$ denote the transition operator of the linear system

$$\begin{aligned} \dot{y}=\left\{ \begin{array}{ccl} -\beta y(t) &{} : &{} t\in [0,1) \hbox { or } 2^{2k+1}\le t\le 2^{2k+2}\,, \\ \gamma y(t) &{} : &{} 2^{2k}\le t\le 2^{2k+1}\,. \end{array}\right. \end{aligned}$$

Hence,

$$\begin{aligned} y(2^{2k+1})&\ge x_0^2\int _{2^{2k}}^{2^{2k+1}} e^{\gamma (2^{2k+1}-s)} e^{-2\alpha s}\,\mathrm {d}s\\&\ge e^{\gamma 2^{2k+1}}\frac{e^{-(2\alpha +\gamma ) 2^{2k}}- e^{-(2\alpha +\gamma ) 2^{2k+1}}}{2\alpha +\gamma }x_0^2\\&\ge \frac{e^{(\gamma -2\alpha )2^{2k}}-1}{2\alpha +\gamma }x_0^2\,, \end{aligned}$$

which proves that $\limsup _{t\rightarrow \infty }y(t)=\infty$ and finishes the proof of this proposition.$\square$

Note that in a forthcoming paper, we will discuss additional conditions on the nonlinearity that guarantee nonlinear stability for systems with negative Bohl spectrum, which include cases where the Sacker–Sell spectrum cannot indicate stability.
